# Distinct functions of transforming growth factor-β signaling in c-MYC driven hepatocellular carcinoma initiation and progression

**DOI:** 10.1038/s41419-021-03488-z

**Published:** 2021-02-19

**Authors:** Haichuan Wang, Pan Wang, Meng Xu, Xinhua Song, Hong Wu, Matthias Evert, Diego F. Calvisi, Yong Zeng, Xin Chen

**Affiliations:** 1 Liver Transplantation Division, Department of Liver Surgery, West China Hospital, Sichuan University, Chengdu, People’s Republic of China; 2 Department of Bioengineering and Therapeutic Sciences and Liver Center, University of California, San Francisco, CA USA; 3 Laboratory of Liver Surgery, West China Hospital, Sichuan University, Chengdu, Sichuan People’s Republic of China; 4 Department of General Surgery, The Second Affiliated Hospital of Xi’an Jiaotong University, Xi’an Jiaotong University, Xi’an, People’s Republic of China; 5 Institute of Pathology, University of Regensburg, Regensburg, Germany

**Keywords:** Cancer microenvironment, Liver cancer, Apoptosis, Growth factor signalling

## Abstract

Dysregulation of transforming growth factor-beta (TGFβ) signaling has been implicated in liver carcinogenesis with both tumor promoting and inhibiting activities. Activation of the c-MYC protooncogene is another critical genetic event in hepatocellular carcinoma (HCC). However, the precise functional crosstalk between c-MYC and TGFβ signaling pathways remains unclear. In the present investigation, we investigated the expression of TGFβ signaling in c-MYC amplified human HCC samples as well as the mechanisms whereby TGFβ modulates c-Myc driven hepatocarcinogenesis during initiation and progression. We found that several TGFβ target genes are overexpressed in human HCCs with c-MYC amplification. In vivo, activation of TGFβ1 impaired c-Myc murine HCC initiation, whereas inhibition of TGFβ pathway accelerated this process. In contrast, overexpression of TGFβ1 enhanced c-Myc HCC progression by promoting tumor cell metastasis. Mechanistically, activation of TGFβ promoted tumor microenvironment reprogramming rather than inducing epithelial-to-mesenchymal transition during HCC progression. Moreover, we identified PMEPA1 as a potential TGFβ1 target. Altogether, our data underline the divergent roles of TGFβ signaling during c-MYC induced HCC initiation and progression.

## Introduction

Hepatocellular carcinoma (HCC) is the most common primary liver malignancy and a leading cause of cancer-related deaths worldwide^1^. Most HCC patients are diagnosed at an advanced stage and current treatments show unsatisfactory therapeutic efficacy^2^. Generally, HCC develops in the context of a diseased liver through a multistep process. Etiologic factors including chronic hepatitis B and C virus infection, aflatoxin exposure, and heavy alcohol consumption contribute to cycles of hepatocyte damage/cell death and compensatory regeneration^3^. These events, together with the progressive accumulation of genetic and epigenetic changes induces a “field defect” in the liver parenchyma, prone to malignant conversion and tumor initiation^4^. The pathophysiology of HCC might be thoroughly divergent during the initiation and progression stages^5^. Thus, there is a need to better delineate the distinct molecular pathways regulating HCC initiation and progression to develop innovative and effective diagnostic and therapeutic approaches.

Dysregulation of the transforming growth factor-beta (TGFβ) signaling is a critical tumorigenic event contributing to hepatocarcinogenesis^6^. TGFβ1, one of the three different homodimer TGFβ isoforms (TGFβ1, TGFβ2 and TGFβ3), is a key member of the TGFβ superfamily^7^. In most cell types, the TGFβ1 ligand binds to type I/II TGFβ receptor (TβRI/II) to induce phosphorylation of mothers against decapentaplegic homolog 2 and 3 (Smad2 and Smad3; activin/TGFβ specific R-Smads)^8^. Activated R-Smads form hetero-oligomeric complexes with a common-partner (co-) Smad, denominated Smad4. The TGFβ1-Smad signaling has been demonstrated to play a critical role in the development of liver cancer^9^. In HCC, TGFβ1 is a bifunctional regulator that either inhibits or promotes hepatocarcinogenesis mainly depending on the tumor stage^10,11^. In particular, TGFβ1 has been shown to be involved in regulating tumor proliferation, epithelial-to-mesenchymal transition (EMT), and tumor microenvironment during HCC progression and metastasis^12^.

Activation of c-MYC is an important oncogenic event during hepatocarcinogenesis. In humans, amplification of c-MYC has been found in ~27% HCC patients^13^. The oncogenic potential of c-Myc has been demonstrated by the finding that *its* overexpression in the mouse liver is sufficient to trigger HCC formation^14,15^. c-MYC has been also shown to directly interact with SMAD2/3 to inhibit TGF-β signaling^16^, thus promoting cell growth and cancer development. However, the crosstalk between c-MYC and TGFβ-SMADs signaling pathways during HCC initiation and progression remains poorly defined.

Here, we investigated the activation of TGFβ signaling in c-MYC amplified human HCC samples. In addition, we determined how the TGFβ cascade modulates c-Myc driven HCC initiation and progression. Our results indicate that TGFβ may function to inhibit HCC initiation but promote metastasis during HCC progression.

## Materials and methods

### Constructs and reagents

The constructs used for mouse injection in this study, including pT3 (Vector), pT3-EF1a-TGFβ1 (mouse), pT3-EF1a-c-Myc (mouse), pCMV, pCMV-Cre, and pCMV-Sleeping Beauty transposase (SB), were previously described^14^. The antisense oligos targeting mouse *Smad2* and *Smad3* were kind gifts from Dr. Simon W. Ro (Kyung Hee University, Seoul, Republic of Korea)^17^. The antisense oligos targeting mouse *Smad4* were designed and generated as previously described^18^. Sequence information for the antisense oligos are: shSmad2, 5′- TGCTGTTGACAGTGAGCGACCGCAGTTAGCTGTGGACTTATAGTGAAGCC-ACAGATGTATAAGTCCACAGCTAACTGCGGGTGCCTACTGCCTCGGA -3′; shSmad3, 5′- TGCTGTTGACAGTGAGCGATAGCTTTGTACTGTATTCTTATAGTGAAGCCACA-GATGTATAAGAATACAGTACAAAGCTAGTGCCTACTGCCTCGGA-3′; shSmad4, 5′- TGCTGTTGACAGTGAGCGCTGAGAATGCACAATCGCCGGATAGTGAAGCCACAGATGTATCCGGCGATTGTGCATTCTCAATGCCTACTGCCTCG -3′. The vector for shSmad plasmid also has EGFP as a reporter for shRNA expression. The EGFP-shSmad sequences were cloned into the pT3-EF1α vector via the Gateway cloning technology (Invitrogen, Carlsbad, CA). Plasmids were purified using the Endotoxin free Maxi Prep Kit (Sigma-Aldrich, St. Louis, MO, USA).

### Mouse treatment and hydrodynamic tail vein gene delivery

Wild-type *FVB/N* mice were obtained from Charles River (Wilmington, MA, USA). Mice used for hydrodynamic injection were 5.5- to 6-week-old. Mice were randomly divided in control and experimental groups and numbers of mice in each group are shown in the related figure legends. No blinding was applied in the study. Hydrodynamic tail vein injections (HTVi) were performed as described^19^. Dosages of the plasmids were as follows: c-Myc 10 μg (or 15 μg for shSmad combination), Mcl-1 10 μg, TGFβ1 40 μg, shLuciferase 30 μg, shSmad2 30 μg, shSmad3 30 μg, shSamd4 30 μg, pCMV 60 μg, pCMV-Cre 60 μg, pT3 60 μg. The dosage for sleeping beauty (SB) was 1/25 of the total oncogene injected. A detailed protocol for HTVi can be accessed at https://pharm.ucsf.edu/xinchen/protocols/hydrodynamic-tail-injection. Mice were monitored by abdominal palpation and euthanized when they developed a high burden of liver tumors, i.e. large abdominal masses. Mice were housed, fed, and monitored in accordance with protocols approved by the Committee for Animal Research at the University of California, San Francisco.

### Murine intrasplenic injection induced liver tumor model

Inducible TGFβ1 (or EGFP) expressing HCC4-4 cells were delivered into the mouse liver through intrasplenic injection via the splenic vein, which joins with the portal vein, as previously described^20^. Briefly, 100 μl ice cold phosphate-buffered saline solution containing 1×10^6^ cells was injected to the spleen of 8-week-old *FVB/N* mouse under general anesthesia. Three days post injection, mice were harvested to evaluate the tumor formation in the spleen at this time point. Doxycycline food (Envigo RMS Inc., Indianapolis, IN) was administrated 3 days after cells implantation. Three mice from each arm (EGFP or TGFβ1) were sacrificed 4 weeks after implantation, and then mice were sacrificed every 3 days, one per group, in parallel. Mouse organs including spleen, liver, lymph node, pancreas, adrenal gland, kidney, colon, diaphragm, abdominal muscles, and lungs were collected for further molecular and histological analyses.

### Protein extraction and Western blot analysis

For total protein extraction, mouse liver tissues and cells were homogenized in M-PER™ Mammalian Protein Extraction Reagent (Cat#78501, Thermo Fisher Scientific) containing the Halt™ Protease Inhibitor Cocktail (Cat#78429, Thermo Fisher Scientific). Subsequently, protein concentration was determined using the Pierce™ Microplate BCA Protein Assay Kit (Cat#23252, Thermo Fisher Scientific). For Western blotting, extracted proteins were boiled in Tris-Glycine SDS Sample Buffer (Bio-Rad Laboratories, Inc., Hercules, CA) for denaturation and subsequently separated by SDS-PAGE, and transferred onto nitrocellulose membranes (Bio-Rad Laboratories, Inc.) by electroblotting. Membranes were blocked in 10% non-fat milk in Tris-buffered saline containing 0.05% Tween-20 for 1 h at room temperature and then incubated with primary antibodies (summarized in Supplementary Table 1) at 4 °C overnight. Membranes were then incubated with horseradish peroxidase-secondary antibody (1:5000; Jackson ImmunoResearch Laboratories Inc., West Grove, PA) at room temperature for 1 h. After appropriate washing, membranes were developed with the Clarity^TM^ Western ECL Substrate (Cat#170-5061, Bio-Rad Laboratories, Inc.) or Clarity^TM^ Max Western ECL Substrate (Cat#170-5062, Bio-Rad Laboratories, Inc.).

### RNA extraction and quantitative real-time polymerase chain reaction (qRT-PCR)

Total mRNA from mouse liver tissues and cells was extracted by using the Quick RNA miniprep kit (Zymo Research, Irvine, CA, USA). Next, mRNA expression was detected by qRT-PCR using the SYBR Green Master Mix (Applied Biosystems, Foster City, CA, USA) in a QuantStudio™ 6 Flex system (Applied Biosystems). Expression of each gene was normalized to the 18 S rRNA gene. Thermal cycling started with an initial hold period at 95 °C for 10 min, and then followed by a three-step PCR program of 95 °C for 15 s, 60 °C for 1 min, and 72 °C for 30 s for a total of 40 cycles. Primers used are listed in Supplementary Table 2.

### Cell culture and in vitro studies

Mouse HCC cell lines (HCC3-4, HCC4-4) were obtained from Dr. Dean Felsher (Stanford University, Palo Alto, CA). The following human HCC cell lines were also used for the in vitro studies: Hep40, HLF, Huh7, and SNU475. Cell lines were authenticated (Genetica DNA Laboratories, Burlington, NC) and tested clear of mycoplasma contamination. Cells were cultured in DMEM medium (Gibco, Grand Island, NY, USA) with 5% fetal bovine serum (Gibco), 100 μg/ml streptomycin, and 100 U/ml penicillin at 37 °C in a 5% CO_2_ humidified incubator. For transfection of EGFP or TGFβ1, pCW57.1-EGFP or pCW57.1-TGFβ1 lentivirus was added to the culture medium when cells reached 50% to 60% confluency in 60 × 15 mm culture dishes. Seventy-two hours later, cells were trypsinized and cultured in 100 × 20 mm dishes in culture medium containing puromycin at the concentration of 1.5 μg/ml for SNU475, 2 μg/ml for HCC3-4 and Huh7, 3 μg/ml for HCC4-4, 5 μg/ml for HLF and 15 μg/ml for Hep40. After 3 days of selection, cells were used for cell migration /invasion and colony formation studies. EGFP and TGF-β1 expression were induced by Doxycycline at a concentration of 4 μg/ml. Proliferation and migration /invasion assays were conducted as described before^14^. For *Pmepa1* knockdown, cells were transfected with siScramble or si*Pmepa1* by using the Thermo Fischer Scientific siRNA Transfection Kit. Cells were harvested for RNA extraction at 48 h after transfection.

### Histology, immunohistochemistry and proliferation and apoptotic indices

Histopathologic examination of the mouse lesions was conducted by a board-certified pathologist and liver expert (M.E.), in accordance with the criteria described previously^18^. Antigen unmasking was achieved by placing the slides in a microwave oven on high for 10 min either in 10 mM sodium citrate buffer (pH 6.0) or 1 mM *ethylenediaminetetraacetic acid* (EDTA; pH 8.5), followed by a 20-min cool down at room temperature. After a blocking step with the 5% goat serum and Avidin-Biotin blocking kit (Vector Laboratories, Burlingame, CA), the slides were incubated with primary antibodies overnight at 4 °C. Slides were then subjected to 3% hydrogen peroxide for 10 min to quench endogenous peroxidase activity and, subsequently, the biotin-conjugated secondary antibody was applied at a 1:500 dilution for 30 min at room temperature. The antibodies used in the experiments are described in Supplementary Table 4. Immunoreactivity was visualized with the Vectastain Elite ABC kit (Cat#PK-6100, Vector Laboratories, Burlingame, CA) and ImmPACT^®^ DAB Peroxidase (HRP) Substrate (Cat#SK-4105, Vector Laboratories, Burlingame, CA). Slides were counterstained with hematoxylin. Proliferation and apoptosis indices were determined in mouse HCC lesions by counting Ki67 and Cleaved Caspase 3 positive cells, respectively. ImageJ 1.8.0 (National Institutes of Health, USA, https://imagej.nih.gov/ij/download.html) and Image Pro Plus 7 (Media Cybernetics, Rockville, MD) software were used for quantification.

### TCGA data analysis

For c-Myc Oncoprint and co-expression analysis, liver hepatocellular carcinoma (TCGA, PanCancer Atlas) data at the public cBioPortal site were utilized. Samples with MYC mutation data (442 patients/samples) were obtained. Genes in Ensembl ID were converted to Entrez ID using the Bioconductor Package Maintainer org.Hs.eg.db, version 3.8.2. Edge R package was used for KEGG analysis. Spearman’s correlation of *PMEPA1* mRNA expression and *TGFB1*, *TGFB2* or *TGFB3* mRNA expression was also assessed. For survival analysis, RNA-seq data, which contain gene expression information of 50 normal liver samples and 370 HCC patients, were obtained from the TCGA datasets. Updated follow-up survival data were downloaded using the R package TCGAbiolinks. The normalized count data calculated by expectation maximization analysis^21^ were incorporated as a matrix in R. With the adoption of an enrichment score cutoff determined by the maximal chi-square method^22^ using R package Maxstat, TCGA HCC samples were categorized into PMEPA1-high and PMEPA1-low signature groups.

### Statistical analysis

The Prism 7.0 software (GraphPad, San Diego, CA) was used to analyze the data, which are presented as Means ± SD. Comparisons between two groups were performed using the two-tailed unpaired *t*-test. Welch correction was applied when necessary. *P*-values < 0.05 were considered statistically significant. Kaplan–Meier survival data were evaluated using a log-rank (Mantel-Cox) test.

## Results

### Activation of the TGFβ pathway in c-MYC amplified human HCC samples

**Fig. 1:**
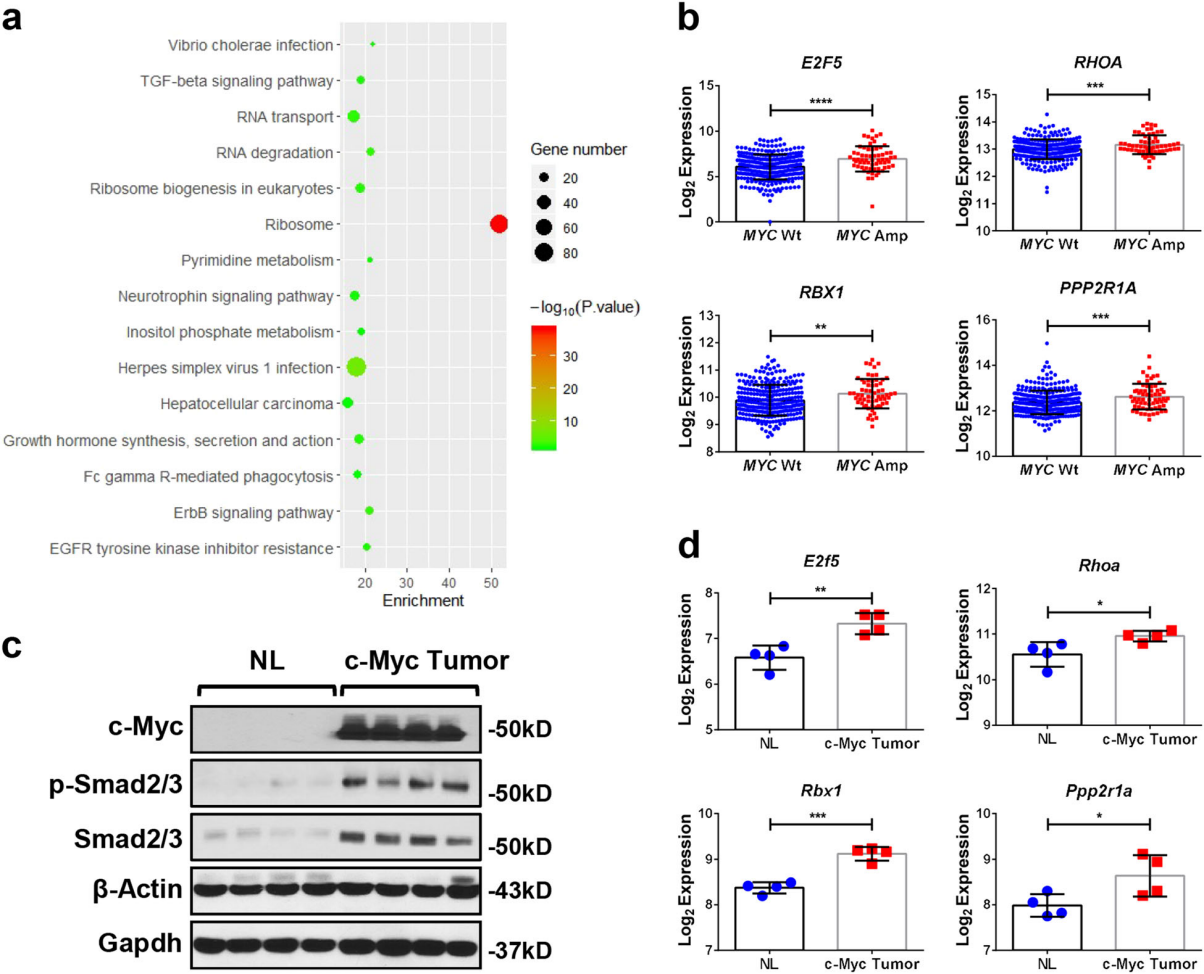
Genetic crosstalk of c-MYC and TGFβ signaling cascades in human HCCs. **a** Kyoto encyclopedia of genes and genomes (KEGG) analysis of statistically significant deregulated genes in HCCs harboring c-MYC amplification, showing a significant gene clustering of TGFβ signaling pathway (*P* = 0.027). **b** Higher mRNA expression of TGFβ downstream target genes (*E2F5*, *RHOA*, *RBX1* and *PPP2R1A*) in c-MYC amplified (MYC Amp; *n* = 64) HCCs than that in c-MYC non-amplified (MYC Wt; *n* = 293) HCCs. Student *t* test was applied for statistical analysis, **P < 0.01, ***P < 0.001, ****P < 0.0001. **c** Western blotting results showing upregulation of p-Smad2/3 in c-Myc murine HCCs (c-Myc Tumor) compared with non-tumorous normal livers (NL). β-Actin and Gapdh were used as loading controls. **d** Microarray analysis of mRNA expressions of TGFβ downstream target genes (*E2f5*, *Rhoa*, *Rbx1*, and *Ppp2r1a*) in c-Myc murine HCCs (c-Myc tumor and non-tumorous normal liver, NL). Student *t* test was applied for statistical analysis. *P < 0.05, **P < 0.01, ***P < 0.001.

To investigate the genes and pathways regulated by c-MYC amplification, we performed bioinformatics analysis of HCC samples from The Cancer Genome Atlas (TCGA) LIHC cohort and identified 2198 genes whose expression levels were deregulated in HCCs harboring c-MYC amplification (Supplementary Table 3). Subsequently, these genes were subjected to Kyoto Encyclopedia of Genes and Genomes (KEGG) analysis. Interestingly, the readout showed a significant TGFβ signaling cluster in c-MYC amplified liver tumors (Fig. 1a). In particular, the expression of TGFβ target genes, such as *E2F5*^23^*, RHOA*^24^*, RBX1*^25^, and *PPP2R1A*^26^, was higher in HCC samples with c-MYC amplification (MYC Amp) than those without amplification (MYC Wt) (Fig. 1b).

To further substantiate this observation, we investigated whether TGFβ signaling is also activated in mouse HCC induced by hydrodynamic tail vein injection (HTVi) of the c-Myc protooncogene^14^. Levels of c-Myc, activated/phosphorylated forms of Smad2/3 (p-Smad2/3) and total Smad2/3 were assessed by Western blot. Upregulation of p-Samd2/3 was found in c-Myc mouse tumors, indicating the activation of the TGFβ signaling (Fig. 1c). Moreover, microarray analysis of c-Myc mouse tumor lesions^14^ revealed significant elevation of TGFβ target genes, including *E2f5*, *Rhoa*, *Rbx1*, and *Ppp2r1a* in mouse HCC (Fig. 1d).

In summary, our data indicate the activation of the TGFβ pathway in c-MYC induced HCC in humans and mice, suggesting a possible functional crosstalk between TGFβ and c-MYC cascades during hepatocarcinogenesis.

### Activation of the TGFβ/SMAD signaling delays HCC development driven by c-Myc overexpression

**Fig. 2:**
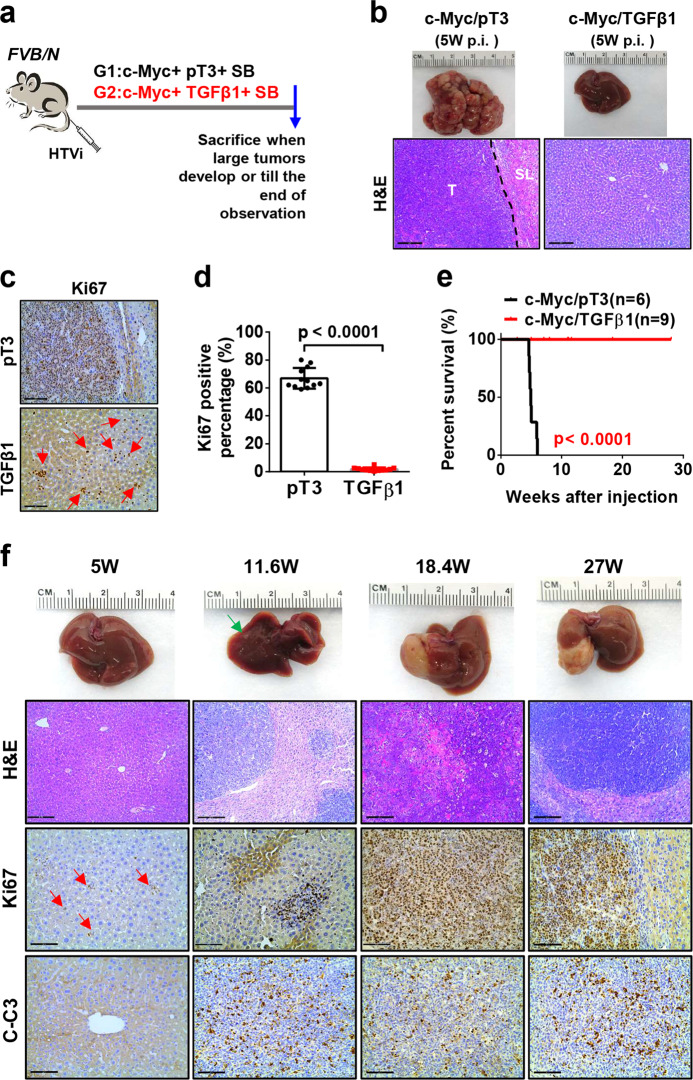
Activation of TGFβ1 delays c-Myc induced hepatocarcinogenesis in mice. **a** Study design. *FVB/N* mice were injected with c-Myc/pT3/SB (*N* = 6) or c-Myc/ TGFβ1/SB (*N* = 9) plasmids. Mice were monitored and sacrificed when moribund. **b** Representative images of tumor and H&E staining in c-Myc/pT3 and c-Myc/TGFβ1 mouse livers at 5 weeks post injection (5 W p.i.). Scale bars: 200μm for 100X. **c** Representative images of Ki67 staining in c-Myc/ pT3 and c-Myc/TGFβ1 mouse livers at 5 weeks post injection (5 W p.i.). Red arrows indicate Ki67+ cells in c-Myc/TGFβ1 group. Scale bars: 100 μm for 200X. **d** Quantification of Ki67+ cells percentages in in c-Myc/pT3 and c-Myc/TGFβ1 mouse livers. Student *t* test was applied for statistical analysis. **e** Survival curve showing that TGFβ1 impaired c-Myc induced hepatocarcinogenesis in mice. Kaplan–Meier method and log-rank test were applied. *P* < 0.0001. **f** Representative images of liver, H&E, Ki67, and Cleaved Caspase-3 (C-C3) immunohistochemical staining in c-Myc/TGFβ1 group at different time points after injection. Green arrow indicates a tumor nodule on the surface of the right lobe of mouse liver. Red arrows indicate Ki67+ cells. Scale bars: 200 μm for H&E, 100 μm for Ki67 and C-C3.

Previous studies suggest that the TGFβ signaling might act either as tumor promoter or tumor suppressor in many cancer types, including HCC^10,15^. As a first step to elucidate the mechanisms whereby TGFβ modulates c-MYC driven HCC development, we determined whether activation of TGFβ accelerates or delays c-Myc HCC initiation in vivo. For this purpose, we co-expressed the TGFβ1 plasmid together with the c-Myc oncogene (c-Myc/TGFβ1) via HTVi. The control group was injected with c-Myc and pT3-EF1α empty vector plasmids (c-Myc/pT3; Fig. 2a). We found that mice in control group rapidly developed HCCs and were moribund by 5 weeks after injection, whereas no preneoplastic and neoplastic lesions were observed in c-Myc/TGFβ1 injected murine livers at the same time point (Fig. 2b). The c-Myc/pT3 HCC lesions demonstrated significant higher proliferation rate than c-Myc/TGFβ1 livers, as determined by anti-Ki67 immunohistochemistry (Fig. 2c, d). Over long time, overexpression of TGFβ1 led to a significant increased overall survival in c-Myc injected mice (Fig. 2e). A few small tumor nodules were observed in c-Myc/TGFβ1 livers at 11.6 weeks post injection, and several larger individual tumor lesions were detected by 18 weeks post injection. Nonetheless, the number of tumor nodules was limited in c-Myc/TGFβ1 mice (Fig. 2f). Moreover, c-Myc/TGFβ1 HCCs exhibited both active proliferation and apoptotic cell death, as indicated by Ki67(+) cells and cleaved caspase-3 (C-C3) (+) cells (Fig. 2f). As expected, the levels of p-Smad2/3 were higher in c-Myc/TGFβ1 tumors than c-Myc/pT3 corresponding lesions (Supplementary Fig. 1), indicating the strong activation of TGFβ signaling in c-Myc/TGFβ1 mouse liver tumors.

**Fig. 3:**
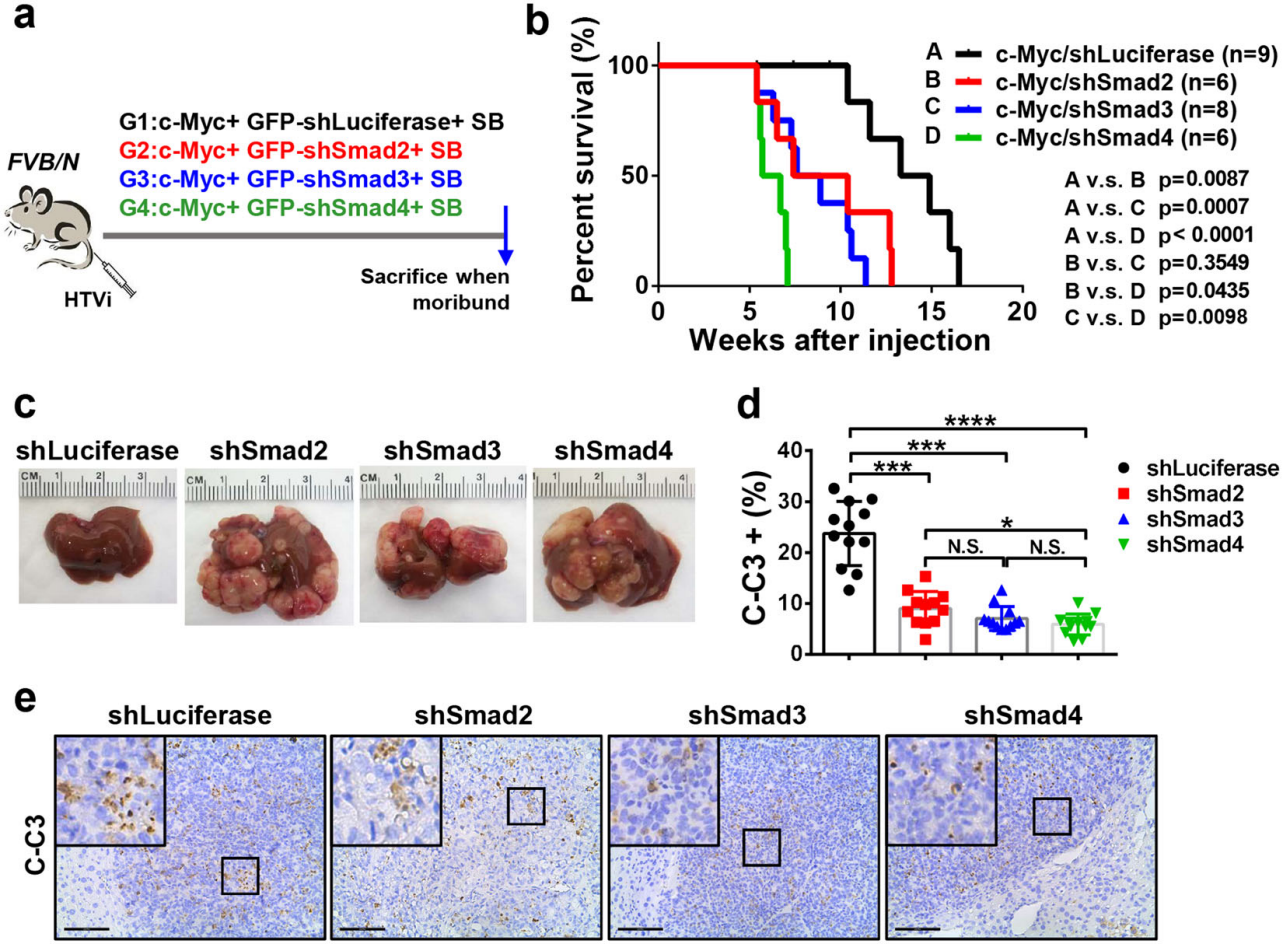
Inhibition of TGFβ-SMAD signaling pathway accelerates c-Myc liver tumorigenesis in mice. **a** Study design. *FVB/N* mice were hydrodynamically injected (HTVi) with c-Myc/GFP-shLuciferase/SB (*n* = 9), c-Myc/GFP-shSmad2/SB (*n* = 6), c-Myc/GFP-shSmad3/SB (*n* = 8) and c-Myc/GFP-shSmad4/SB (*n* = 6) plasmids, respectively. Mice were monitored and sacrificed when moribund. **b** Survival curve showing that inhibition of Smad2, Smad3 or Smad4 accelerates c-Myc induced hepatocarcinogenesis in mice. Kaplan–Meier method and log-rank test were applied. *P*-values were shown in the figure. **c** Representative images of tumors in the four groups at 5 weeks post injection (p.i.). **d** Comparison of cleaved caspase 3 (C-C3) positive cells percentages in the four groups. Student *t-*test was applied between each 2 groups for statistical analysis, *P < 0.05, ***P < 0.001, ****P < 0.0001, N.S., no significance. **e** Representative images of cleaved caspase 3 (C-C3) immunohistochemical staining in c-Myc/shLuciferase (shLuciferase), c-Myc/shSmad2 (shSmad2), c-Myc/shSmad3 (shSmad3) and c-Myc/shSmad4 (shSmad4) mouse HCCs. Scale bars: 200 μm.

Next, we investigated whether TGFβ1 dependent suppression of c-Myc driven hepatocarcinogenesis was mediated by the Smad2/3 and Smad4 complexes. Thus, we generated short hairpin RNAs (shRNAs) targeting *Smad2*, *Smad3*, and *Smad4* into pT3-EF1α vector downstream of the *GFP* sequence. Subsequently, shSmad2, shSmad3, or shSmad4 plasmids were co-expressed with c-Myc in the mouse liver via HTVi. Additional mice were injected with c-Myc together with shRNA against Luciferase (shLuciferase) as control (Fig. 3a). Importantly, silencing of Smad2, Smad3 or Smad4 significantly accelerated c-Myc HCC formation (Fig. 3b). By 5 weeks after injection, tumor nodules were obvious in shSmad2, shSmad3, and shSamd4 co-injected mice, while neoplastic lesions were rarely detected in control shLuciferase injected mice (Fig. 3c). It is worth to note that while silencing of either *Smad2* or *Smad3* led to accelerated c-Myc tumor growth, silencing of Smad4 demonstrated the most significant tumor acceleration phenotype (Fig. 3b and 3c). This observation is consistent with the fact that Smad2 and Smad3 may be functional redundant, whereas Smad4 is the unique subunit in the Smad complex downstream of TGFβ signaling^27^. Histologically, a small cluster of HCC tumor cells with lower proliferation rate, as indicated by Ki67 staining, was detected in shLuciferase injected mice (Supplementary Fig. 2a). The expression of shSmads or shLuciferase in HCC lesions was validated via immunofluorescence staining of GFP (Supplementary Fig. 2b). Altogether, the present data indicate that silencing of Smad2/3/4 accelerates c-Myc driven HCC initiation.

Next, we investigated the molecular mechanisms underlying the observed phenotypes. Thus, we analyzed tumor cell proliferation and apoptosis rates in c-Myc/shLuciferase and c-Myc/shSmads mouse HCC. Intriguingly, cell proliferation showed no difference between c-Myc/shLuciferase and c-Myc/shSmads liver tumors, as indicated by Ki67 index (Supplementary Fig. 2c). In contrast, cell apoptosis was dramatically hampered in shSmad2, shSmad3, and shSmad4 HCC lesions, as measured by cleaved caspase 3 index (Fig. 3d and e).

Altogether, these findings indicate that the TGFβ pathway might modulate c-Myc HCC initiation by regulating c-Myc induced apoptosis. Specifically, inhibition of TGFβ might prevent c-Myc induced cell death, leading to accelerated tumor development, whereas overexpression of TGFβ1 might facilitate apoptosis, resulting in the delay of HCC formation. If this hypothesis is correct, one could predict that co-expression of an anti-apoptotic mediator, such as Mcl-1, would rescue TGFβ1 dependent tumor inhibition phenotype, i.e., overexpression of TGFβ1 would not be able to suppress tumor development induced by c-Myc and Mcl-1 oncogenes in the liver. To test the hypothesis, we co-delivered c-Myc, Mcl-1, and TGFβ1 (c-Myc/Mcl-1/TGFβ1) plasmids to the mouse liver. Additional mice were injected with c-Myc, Mcl-1, and pT3-EF1α empty vector (c-Myc/Mcl-1/pT3) as control (Supplementary Fig. 3a). Noticeably, consistent with our hypothesis, overexpression of TGFβ1 did not affect liver tumor development in c-Myc/Mcl-1 mice (Supplementary Fig. 3b–d).

In summary, the present data show that overexpression of TGFβ1 significantly delays c-Myc dependent hepatocarcinogenesis, whereas suppression of the TGFβ/Smad pathway accelerates c-Myc HCC initiation by hindering c-Myc induced apoptosis.

### TGFβ1 promotes c-Myc liver tumor metastasis

**Fig. 4:**
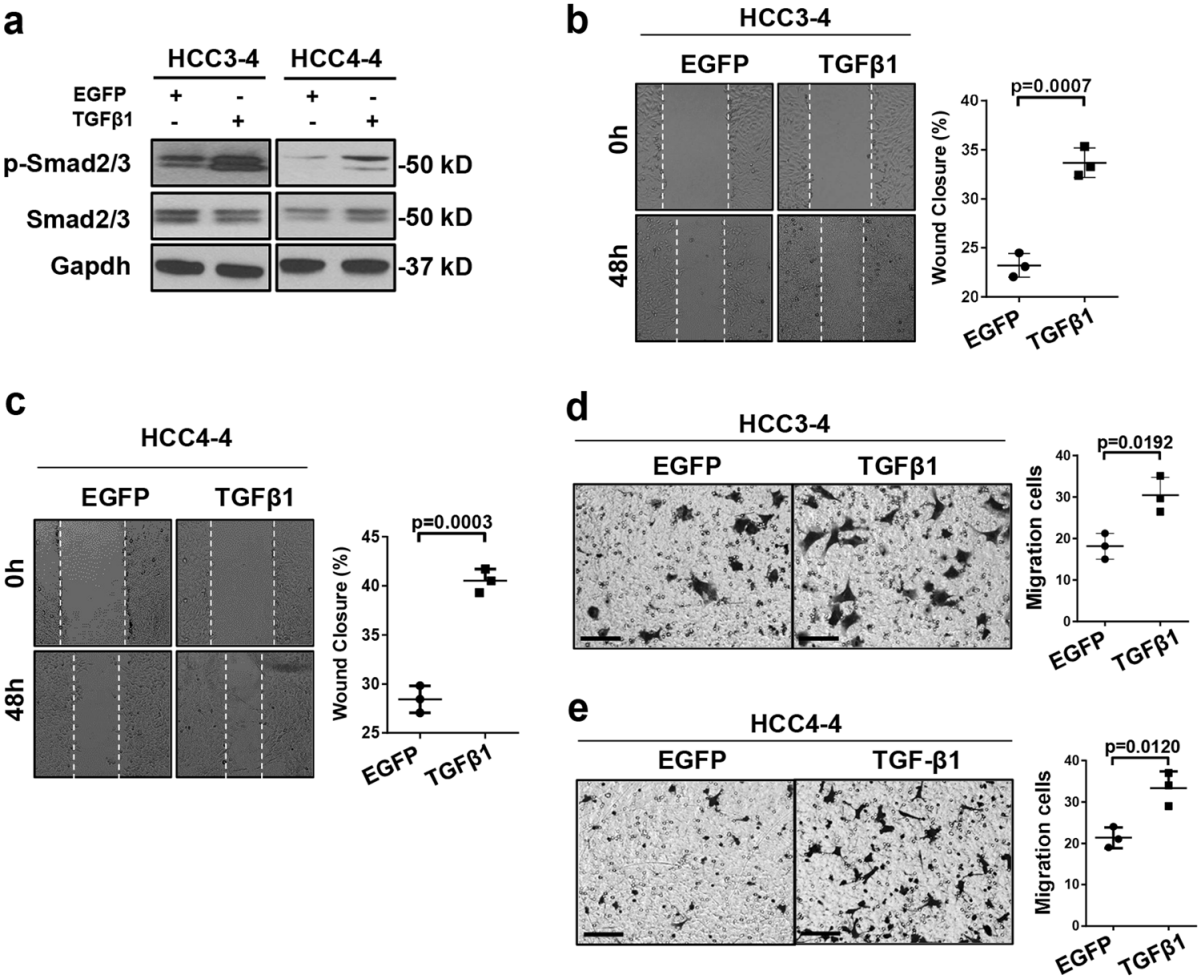
TGFβ1 activation promotes cell migration and invasion in c-Myc murine tumor derived HCC cell lines. **a** Western blotting results showing activation of Smad2/3 (p-Smad2/3) in TGFβ1 overexpressing HCC3-4 and HCC4-4 cells. Gapdh was used as a loading control. **b**, **c** Representative images and quantifications of cell healing assay at 0-hour (0 h) and after 48 hours (48 h) treatment for inducing TGFβ1 (*N* = 3) or EGFP (*N* = 3) expression in **b** HCC3-4 and **c** HCC4-4 cell lines. **d, e** Representative images and quantification of transwell migration assay 48 h after treatment for inducing TGFβ1 (*N* = 3) or EGFP (*N* = 3) expression in (d) HCC3-4 and (e) HCC4-4 cell lines. Student *t* test was applied for statistical analysis. *P* values were as shown. Experiments were conducted three times.

Our study points to a tumor suppressor role of the TGFβ/Smad cascade during tumor initiation. Since the TGFβ pathway possesses context-dependent tumor inhibitory and tumor promoting activities^10,15^, we sought to investigate the role of TGFβ on c-Myc driven tumor progression. For this purpose, we applied murine HCC cell lines, namely HCC3-4 and HCC4-4 cells, which are derived from c-Myc mouse HCCs^28^. Specifically, HCC3-4 and HCC4-4 cell lines were transfected either with the doxycycline inducible TGFβ1 expression plasmid or with the EGFP construct (as control). As expected, following doxycycline administration, HCC3-4 and HCC4-4 cells exhibited higher levels of p-Smad2/3 when transfected with TGFβ1, indicating the activation of TGFβ-Smad signaling upon TGFβ1 overexpression (Fig. 4a). Activation of the TGFβ pathway did not affect c-Myc HCC cell growth, as determined by cell proliferation and colony formation assays (Supplementary Fig. 4). In contrast, activation of the TGFβ signaling significantly promoted c-Myc tumor cell migration, as measured by cell wound healing assay (Fig. 4b, c), and cell invasion, as evaluated by transwell migration assay (Fig. 4d, e).

**Fig. 5:**
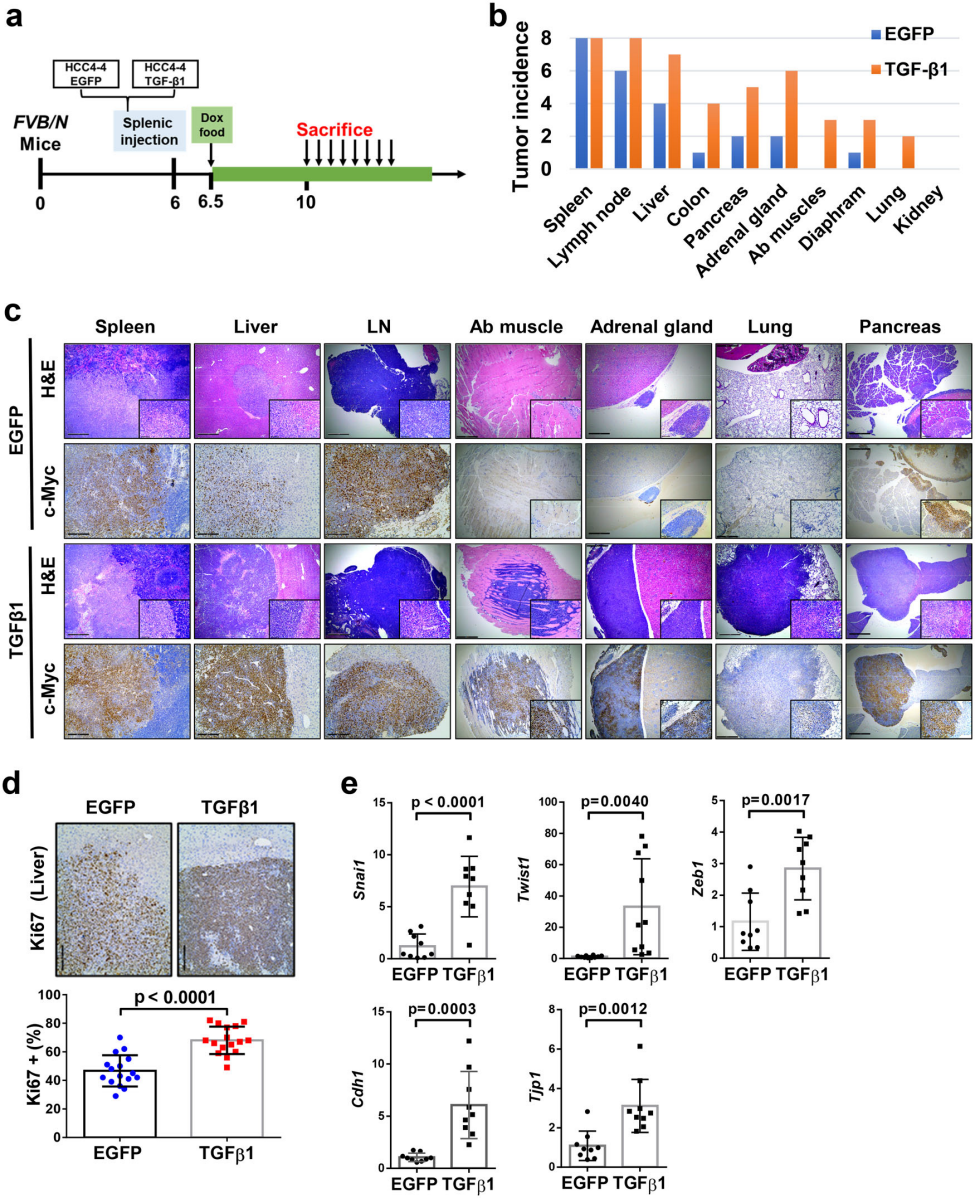
TGFβ1 activation promotes c-Myc positive tumor cells progression and metastasis in vivo. **a** Study design. Inducible TGFβ1 (or EGFP) expressing HCC4-4 cells were delivered into mouse liver through intrasplenic injection. Food containing doxycycline (Dox food) was administered 3 days after implantation to induce TGFβ1 or EGFP expression. Three mice from each arm (EGFP or TGFβ1) were sacrificed 4 weeks after implantation, and then mice were sacrificed every 3 days one per group in parallel. **b** Numbers of mice showing tumors in each organ at the time of sacrifice from EGFP (*N* = 8) and TGFβ1 (*N* = 8) groups. The *Y* axis (tumor incidence) represents the number of mice from the EGFP or TGFβ1 group displaying neoplastic lesions in each organ. Tumors were examined macroscopically and microscopically by H&E staining. **c** Representative images of H&E and c-Myc immunohistochemical staining in EGFP and TGFβ1 groups in different organs. Black boxes on right lower corner denote enlarged views for better visualization. Scale bars: 200 μm for H&E, 100 μm for c-Myc. **d** Representative images of Ki67 immunohistochemical staining and quantification of Ki67 positive cell percentage in liver tumors from both groups. Student *t* test was applied for statistical analysis. *P* < 0.0001. Scale bars: 100 μm. **e** mRNA expression of TGFβ downstream target genes (*Snai1 Twist1*, and *Zeb1*) and epithelial marker genes (*Cdh1 and Tjp1)* in EGFP or TGFβ1 activated murine liver tumors. Student *t*-test was applied for statistical analysis.

Next, to delineate the role of TGFβ1 signaling in modulating the progression, especially metastasis, of c-Myc induced mouse HCCs in vivo, we employed the intrasplenic injection tumor model. Specifically, inducible TGFβ1 (or EGFP) expressing HCC4-4 cells were delivered into the mouse liver through intrasplenic injection via the splenic vein, which joins with the superior mesenteric vein to become the portal vein^20^. It is important to note that, consistent with previous findings^29^, microscopic lesions could be clearly visualized in the mouse spleen by 3 days after injection (Supplementary Fig. 5), suggesting that the tumors have developed at this time point. By administering doxycycline with the food, TGFβ1 expression is induced in the mouse HCC, thus allowing the investigation of TGFβ1 role in tumor progression. Three mice from each arm (EGFP or TGFβ1) were sacrificed 4 weeks after implantation as baseline. Mice were subsequently sacrificed every 3 days, one per group, in parallel (Fig. 5a). Mouse organs, including spleen, liver, lymph nodes, pancreas, adrenal glands, kidney, colon, diaphragm, abdominal muscles, and lungs were collected and subjected to H&E staining for tumorigenesis analysis. As expected, all mice eventually developed tumors in the spleen, and no difference in terms of tumor burden on the spleen was noted. The results were consistent with in vitro analyses showing that overexpression of TGFβ1 does not significantly affect tumor growth. Noticeably, TGFβ1 activation promoted HCC metastasis, as indicated by higher tumor incidence in the liver, abdominal lymph nodes as well as other organs (Fig. 5b). Of note, liver tumor nodules were detected as early as 4 weeks after tumor cell implantation in the TGFβ1 group, whereas no tumor was detected in the control group. Presence of c-Myc positive tumor cells was confirmed by immunohistochemistry for c-Myc protein (Fig. 5c). Further analysis revealed that the proliferation rate was significantly increased in livers with activated TGFβ1, as indicated by a higher Ki67(+) cell percentage (Fig. 5d).

Overall, our data show that conditional activation of TGFβ1 in c-Myc positive HCC cells contributes to tumor metastasis in vitro and in vivo.

### EMT might be dispensable for TGFβ to promote c-Myc HCC metastasis

Next, we focused on the liver lesions induced by the intrasplenic injection method to further explore the function of the TGFβ1 cascade in c-Myc HCC progression, especially metastasis. Previously, it has been reported that the TGFβ signaling induces tumor progression by regulating epithelial to mesenchymal transition (EMT)^30,31^ and/or modulating the tumor microenvironment (TME)^32,33^. First, we tested the hypothesis that TGFβ promotes c-Myc HCC progression and metastasis by inducing EMT. At the molecular level, TGFβ downstream target genes (*Snail*, *Twist1*, and *Zeb1*) were found to be upregulated in the lesions. Nonetheless, overexpression of TGFβ1 led also to the upregulation of epithelial genes (*Cdh1* and *Tjp1*) (Fig. 5e). A similar mRNA trend upon TGFβ1 overexpression was also observed in vitro using HCC3-4 and HCC4-4 cell lines (Supplementary Fig. 6). Consistently, using immunofluorescence, no overlapping staining pattern for E-cadherin and Vimentin in c-Myc/Mcl-1/TGFβ1 murine HCCs was detected (Supplementary Fig. 7), which further substantiated the absence of EMT in TGFβ1 activated c-Myc tumors.

Next, we investigated whether TGFβ may modulate EMT in human HCC samples. We reasoned that loss of epithelial cell marker expression was a better indicator than the gain of expression of mesenchymal markers in bulk RNASeq datasets, because one could not distinguish whether the increased mesenchymal marker expression is due to increased fibroblast cells within tumor samples or EMT. We searched TCGA LIHC dataset and analyzed whether TGFβ ligand isoforms, including TGFβ1, TGFβ2, and TGFβ3, demonstrate a negative correlation with epithelial markers, such as *CDH1* and *TJP1*. We did not observe any negative correlation between TGFβ isoforms and *CDH1* or *TJP1* mRNA expression in TCGA LIHC samples (Supplementary Fig. 8).

Altogether, the present findings suggest that TGFβ induced c-Myc murine HCC metastasis might be independent of the EMT program.

### TGFβ induced TME adaptiveness is required for c-Myc mouse HCC progression

Previous studies have shown that TGFβ may modulate TME and promote pro-inflammatory responses^34,35^. To test whether these events might contribute to TGFβ induced accelerated c-Myc HCC progression, we first examined the changes of tumor related immune milieu in EGFP and TGFβ1 expressing c-Myc liver tumors. Thus, immunohistochemical analysis for the lymphocyte marker Cd45 and the macrophage marker F4/80 was performed. Intriguingly, TGFβ1 activation enhanced lymphocyte infiltration while reduced macrophages in c-Myc liver tumors (Supplementary Fig. 9). These results suggest that TGFβ1 might be involved in the regulation of the tumor microenvironment in the c-Myc murine model.

**Fig. 6:**
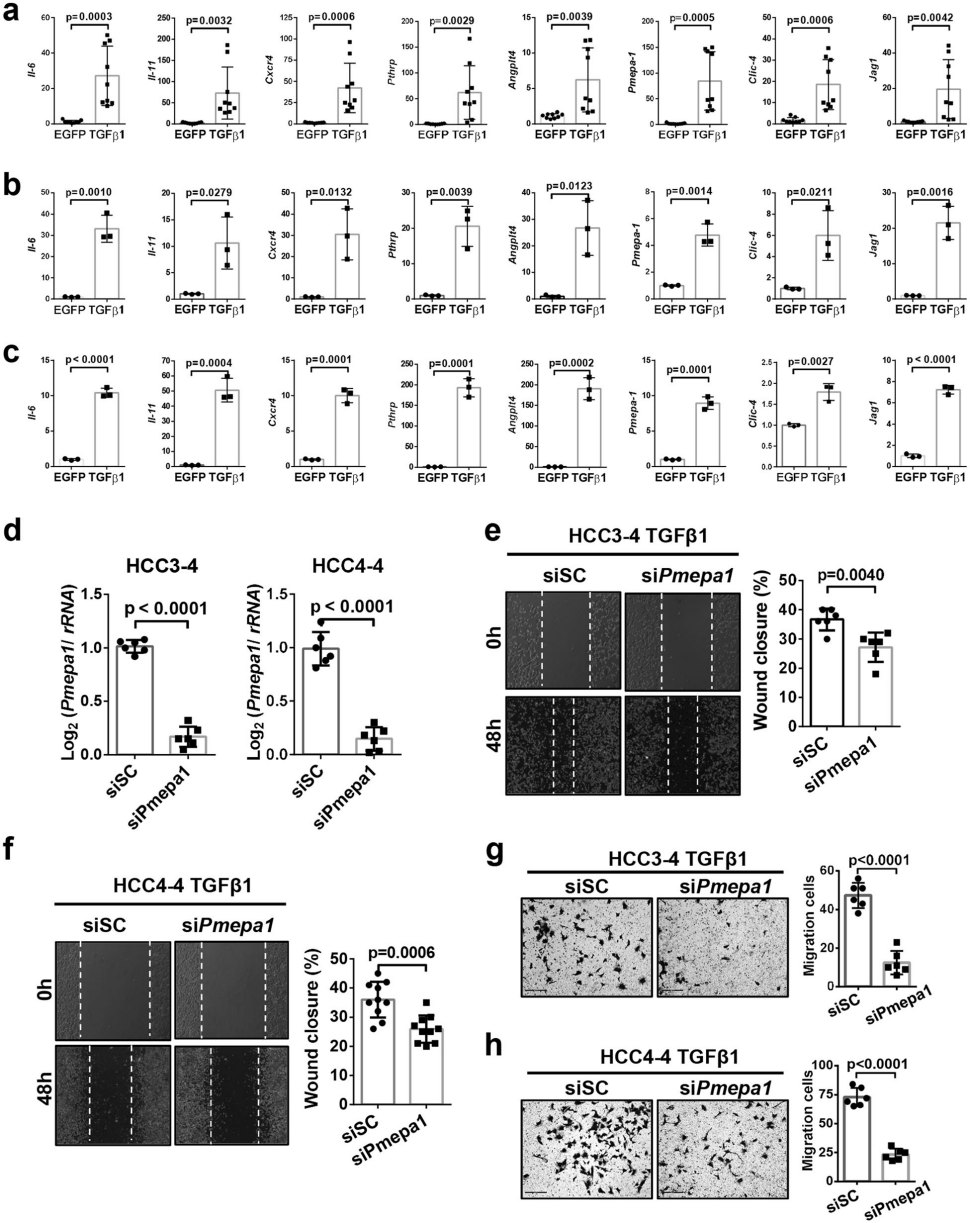
TGFβ1 activation induces tumor microenvironment (TME) reprogramming in c-Myc murine HCCs. **a** Upregulation of TGFβ downstream target genes related to TME reprogramming in EGFP or TGFβ1 activated liver tumors. Student *t* test was applied for statistical analysis. **b, c** Upregulation of TGFβ downstream target genes related to TME in EGFP or TGFβ1 activated **b** HCC3-4 cell lines and **c** HCC4-4 cell lines. Student *t* test was applied for statistical analysis. **d** Quantitative RT-PCR results showing downregulation of *Pmepa1* in TGFβ1 activated HCC3-4 and HCC4-4 cell lines after si*Pmepa1* transfection (*N* = 6 replicates in each group). Student *t* test was applied for statistical analysis. **e, f** Representative images and quantification of cell healing assay at 0-hour (0 h) and after 48 hours (48 h) treatment following TGFβ1 overexpression as well as scrambled siRNA (siSC) or si*Pmepa1* transfection. Student *t*-test was applied for statistical analysis. *P*-values were as shown. Experiments were conducted three times. **g, h** Representative images and quantification of transwell assay 48 h after treatment following TGFβ1 overexpression as well as scrambled siRNA (siSC) or si*Pmepa1* transfection. Student *t-*test was applied for statistical analysis. *P-*values were as shown. Experiments were conducted three times.

Subsequently, we performed quantitative real-time PCR analysis of c-Myc HCC tissues with or without TGFβ1 overexpression both in vivo and in vitro. Intriguingly, we discovered that TGFβ1 activated c-Myc murine liver tumors exhibited the upregulation of pro-inflammatory cytokine genes (Interleukin 6, *Il-6*; and Interleukin 11, *Il-11*), chemokine related genes (chemokine receptor type 4, *Cxcr4*), and genes associated with tumor microenvironment (parathyroid hormone-related protein, *Pthrp*; angiopoietin-like 4, *Angptl4*; transmembrane prostate androgen-induced protein 1, *Pmepa1*; chloride intracellular channel 4, *Clic4*; and Jagged-1, *Jag-1*) (Fig. 6a). Consistently, activation of the TGFβ signaling also significantly activated the expression of these genes in vitro (Fig. 6b, c).

Among the genes induced by TGFβ1, transmembrane prostate androgen-induced protein 1 (*Tmepa1* or *Pmepa1*) has been reported to be a biomarker for the TGFβ inhibitor Galunisertib in HCC^36^. Thus, we tested whether blocking Pmepa1 was able to modulate HCC progression. Knocking down of mouse *Pmepa1* was achieved by specific small interfering RNAs, and siRNA targeting efficacy was first validated by qRT-PCR (Fig. 6d). Functional analysis revealed that cell migration and invasion were significantly inhibited when knocking down *Pmepa1* in TGFβ activated HCC3-4 and HCC4-4 cell lines (Fig. 6e–h).

**Fig. 7:**
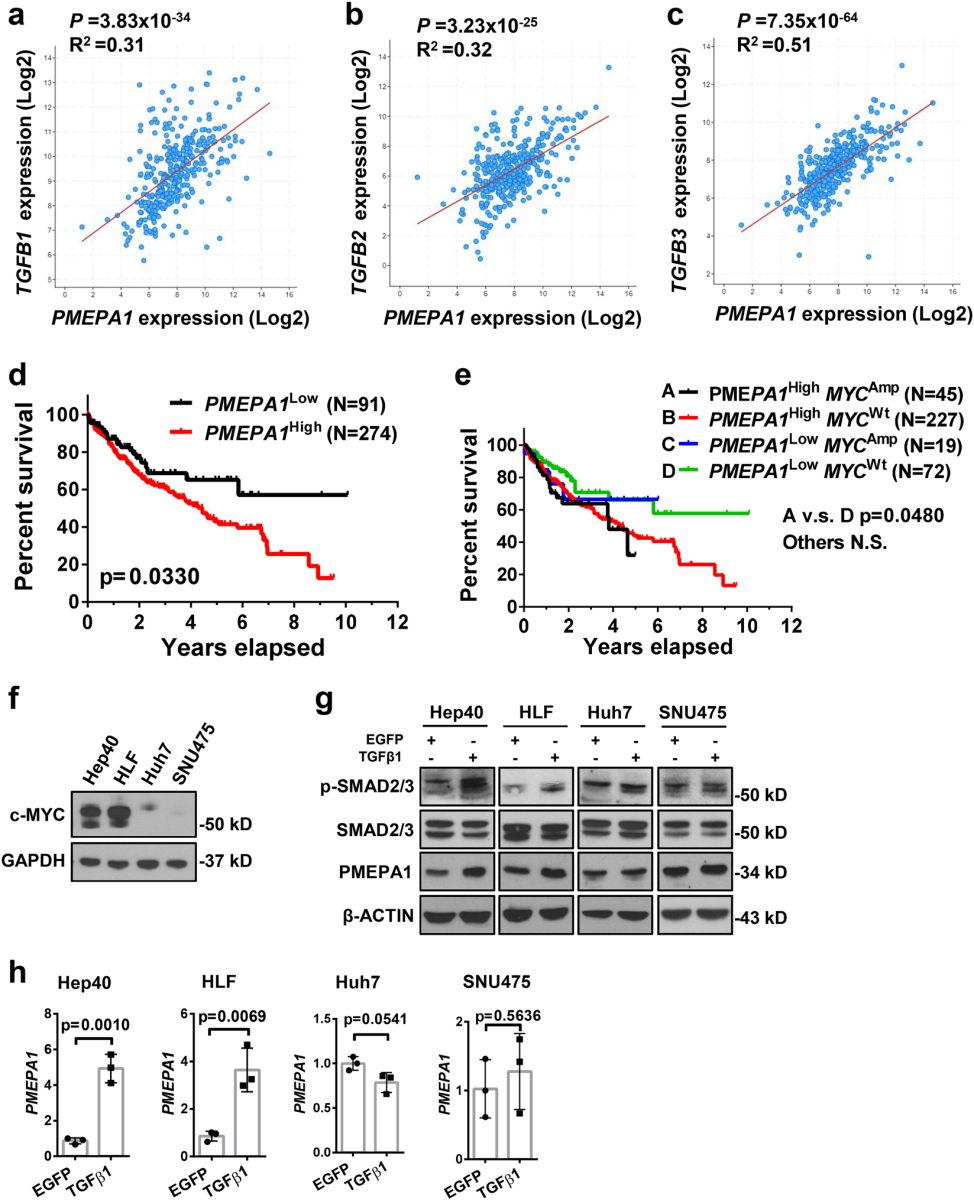
*PMEPA1* expression correlates with TGFβ expression and poor survival outcome in human HCC patients. **a**–**c** Spearman’s correlation of *PMEPA1* mRNA expression and **a** *TGFβ1*, **b** *TGFβ2* and **c** *TGFβ3* mRNA levels in human HCCs showing a strong significant positive correlation. Images were obtained from the public cBioPortal website (https://www.cbioportal.org/). **d** High PMEPA1 expression (*N* = 274) showed a poor survival outcome when compared with low PMEPA1 expression (*N* = 91) in human HCC patients. Kaplan–Meier method and log-rank test were applied. *P* = 0.033. **e** High PMEPA1 expression and c-MYC amplified patients (MYC ^Amp^; *N* = 45) showed a poor survival outcome when compared with low PMEPA1 expression and MYC non-mutant (MYC ^Wt^; *N* = 72) HCC patients. Kaplan–Meier method and log-rank test were applied. *P* = 0.048. **f** Western blot analysis showing the expression of c-MYC in human HCC cells. GAPDH was used as a loading control. **g** Western blot analysis showing the expression of p-SMAD2/3, SMAD2/3, and PMEPA1 in EGFP and TGFβ1 transfected human HCC cells. β-ACTIN was used as a loading control. **h** mRNA expression of *PMEPA1* gene in EGFP or TGFβ1 transfected human HCC cells. Student *t* test was applied for statistical analysis.

In human HCCs, *PMEPA1* mRNA level was found to be significantly correlated with *TGFβ1*, *TGFβ2*, and *TGFβ3* mRNA expression (Fig. 7a–c), further suggesting the tight correlation between TGFβ signaling and PMEPA1 during liver carcinogenesis. High *PMEPA1* expression was also associated with a poor survival outcome (Fig. 7d). Furthermore, *PMEPA1* and *c-MYC* together might also serve as prognosis marker for HCC, as *c-MYC* amplification HCCs with high *PMEPA1* expression showed the worst overall survival outcome (Fig. 7e). Furthermore, we found that TGFβ1 induced PMEPA1 upregulation in c-MYC high expressed human HCC cell lines (Hep40 and HLF)^37^, but not in c-MYC low expressed human HCC cell lines (Huh7 and SNU475; Fig. 7f–h), suggesting that PMEPA1 may be a target of TGFβ1 in the context of c-MYC HCC.

Altogether, the present data indicate a crucial contribution of TGFβ1 related tumor microenvironment reprogramming in modulating c-Myc HCC progression. PMEPA1 might represent one of the relevant targets induced by TGFβ1 during this process.

## Discussion

HCC frequently occurs after liver chronic damage in a multistep process, starting from hepatocyte compensatory proliferation to the sequential formation of dysplastic nodules, early tumors, and progressed HCC. The TGFβ pathway has been reported to be involved in both HCC initiation and progression stages^38,39^, and members of this cascade have been shown to be mutated in ~40% HCC cases^6^. Moreover, the mRNA levels of TGFβ as well as its downstream effectors, the SMAD family members, are frequently overexpressed in HCC (Supplementary Fig. 10). Nevertheless, the role of the TGFβ signaling during HCC development remains controversial and the complex function of TGFβ signaling might be context- and oncogene-dependent^40^. Amplification of c-MYC occurs in a significant subset of human HCCs, and the functional interplay of c-MYC with the TGFβ signaling remains undefined. Thus, our study provides for the first time a comprehensive investigation of TGFβ1 during c-MYC liver tumor development and progression.

**Fig. 8:**
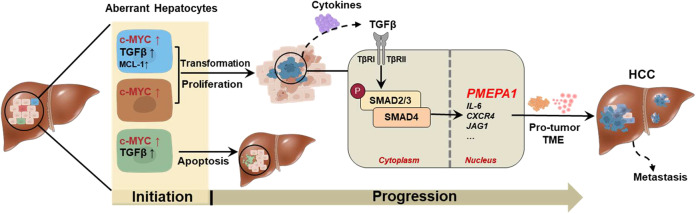
Scheme showing the role of TGFβ-SMAD signaling in the regulation c-Myc induced hepatocarcinogenesis. Abbreviation: TME, tumor microenvironment.

A major finding of the current study is that TGFβ1 suppresses HCC initiation in the context of c-Myc activation (Fig. 8). By using hydrodynamic transfection approaches, we provided comprehensive perception of the role of TGFβ1/SMAD pathway in regulating HCC initiation in vivo. Specifically, overexpression of TGFβ1 hampers hepatocarcinogenesis driven by c-Myc. Consistently, shRNA mediated silencing of *Smad2*, *Smad3*, or *Smad4*, or overexpression of inhibitory *Smad7* (data not shown) led to accelerated HCC onset in c-Myc mice. Of note, the mixture of c-Myc and shRNA expressing plasmids induced murine HCC later than c-Myc in combination with other plasmids. This event might be due to the high diluting efficiency of shRNA plasmids and the high cell death level induced by shRNA expressed plasmid. Our finding is consistent with the previous study by Moon *et al*., showing that TGFβ inhibition promotes RAS mediated oncogenesis in c-Myc overexpressing livers^17^. Mechanistically, we showed that activation of the TGFβ cascade triggers c-Myc induced apoptosis, leading to the suppression of tumor initiation. Indeed, when *c-MYC* activation/overexpression was coupled to overexpression of the anti-apoptotic gene *Mcl-1*, the pro-apoptotic function of TGFβ1 was bypassed, ultimately resulting in tumor formation.

It is worth to note that tumor lesions, although with a longer latency, still developed in c-Myc/TGFβ1 injected mice. Nonetheless, in contrast to the high number of HCC lesions in the c-Myc HCC model, tumor number in c-Myc/TGFβ1 mice was extremely low. Intriguingly, the eventual liver tumor nodules developing in c-Myc/TGFβ1 mice expressed higher levels of multiple anti-apoptosis molecules, including B-cell lymphoma-extra-large (Bcl-xl) and Mcl-1 (Supplementary Fig. 11). Considering the limited tumor nodules in c-Myc/TGFβ1 HCC model, one plausible explanation is that a small percentage of c-Myc injected hepatocytes were able to induce the expression of these anti-apoptotic genes, thus escaping TGFβ1 induced apoptosis. Clearly, additional studies are required to investigate the precise mechanisms whereby c-Myc overexpressing hepatocytes induce the expression of these anti-apoptotic genes, and whether this phenotype depends on secondary mutations occurring in tumor nodules or due to additional epigenetic modifications.

Another finding of our investigation is that TGFβ1 modulates c-Myc HCC metastasis. The studies were performed using murine c-Myc derived HCC cell lines, and both in vitro migration/invasion and in vivo metastasis assays were conducted. An alternative approach to the usage of cell lines would consist in the direct induction of TGFβ1 in c-Myc HCC mouse lesions. This could be achieved via generating the pT3-TRE-TGFβ1 plasmid, and injecting the plasmid into mice together with c-Myc and sleeping beauty transposase constructs via HTVi into rtTA transgenic mice^41^. Subsequently, mice would be allowed to develop tumors, and TGFβ1 could be induced in tumor bearing mice via feeding the mice a doxycycline diet. This approach would provide further support for the role of TGFβ1 in regulating c-Myc HCC progression in vivo. Importantly, such approach will be necessary to investigate TGFβ1 in regulating tumor progression when HCC cell lines are not available, such as in c-Met/β-Catenin induced mouse HCC.

Mechanistically, we found that TGFβ1 regulates tumor progression via tumor microenvironment reprogramming rather than by inducing EMT. Although previous studies suggested that EMT is a major cellular mechanisms in TGFβ pro-tumorigenic function^42^, our results show the lack of EMT induction in TGFβ1 activated c-Myc HCCs at the molecular level. Consistently, in human HCC samples, we discovered that TGFβ related downstream EMT targets such as *CREBBP*^43^*, EP300*^44^*, APC*^45^*, SKIL*^46^, and *MAP2K1 (MEK1)*^47^ are not overexpressed in c-MYC amplified tumor samples based on TCGA LIHC data analysis (Supplementary Fig. 12). We found instead the significant upregulation of inflammatory cytokines and angiogenesis related genes in vivo and in vitro when TGFβ1 was overexpressed in c-MYC HCC cells. Therefore, TGFβ regulated tumor microenvironment reprogramming might be the main mechanism whereby TGFβ promotes c-MYC HCC progression.

Our data also suggest that the TGFβ cascade may play a key role in modulating immune cells in HCC. Currently, immunotherapies, especially checkpoint inhibitors, have shown great promise^48^ as the first line treatment strategy against HCC. For instance, the IMBRAVE-150 clinical trial demonstrated the superior efficacy of anti-VEGF and anti-PDL1 antibodies for the treatment of advanced stage HCC than that of Sorafenib^48^. Nonetheless, the clinical trial also showed that most of the patients progressed under the combination immunotherapy^48^. Activated TGFβ has been proposed as a possible mediator of immunotherapy resistance^49^. Accordingly, a recent study from our group showed that c-Myc mouse HCCs were resistant to anti-PDL1 antibody treatment^50^. Thus, it would be of high importance to investigate whether the activated TGFβ signaling contributes to anti-PDL1 resistance in c-Myc mouse HCC. Furthermore, the combination of anti-PDL1 antibodies with TGFβ pathway inhibitors, such as Galunisertib^51^, should be tested and may be effective for HCC treatment. The c-Myc mouse model may represent an excellent preclinical tool to validate this hypothesis.

In this manuscript, we discovered *PMEPA1* as a potential target of the TGFβ cascade in c-MYC HCC. PMEPA1 is an androgen-regulated protein highly expressed in various solid tumors, including HCC^52^. PMEPA1 is known to play an important role downstream of the TGFβ signaling pathway in prostate^53^ and breast cancers^54^. In a comprehensive genome-wide mouse HCC microarrays study, *PEMPA1* was identified as a classifier for HCC with a late TGFβ signature, which accurately predicted liver metastasis^55^. However, the function of PMEPA1 in HCC has not been established to date. Here, we show that high *PMEPA1* expression correlates with poor survival outcome in human HCCs and targeting *Pmepa1* inhibits TGFβ1 activated c-Myc murine HCC cells migration. Moreover, we found that *PMEPA1* mRNA expression is upregulated in human HCC samples (Supplementary Fig. 13), and its level correlates with *TGFβ1*, *TGFβ2*, and *TGFβ3* mRNA expression in TCGA liver cancer samples (Fig. 7a). These findings are consistent with the data from Cao et al.^36^ using an independent HCC cohort. As TGFβ likely regulates tumor progression via multiple genes/pathways, and PMEPA1 may only represent one of these genes, further studies are needed, especially in vivo, to delineate the functional contribution of PMEPA1 in HCC progression. In the current study, we found that TGFβ1 activation induced upregulation of PMEPA1 in c-MYC high expressed human HCC cell lines, but not in c-MYC low expressed human HCC cell lines. The results support the hypothesis that PMEPA1 may be a major TGFβ effector and serve as a target for c-MYC activated liver cancer treatment.

## Supplementary information

Supplementary Table 1

Supplementary Table 2

Supplementary Table 3

Supplementary Figure Legends

Supplementary Fig. 1

Supplementary Fig. 2

Supplementary Fig. 3

Supplementary Fig. 4

Supplementary Fig. 5

Supplementary Fig. 6

Supplementary Fig. 7

Supplementary Fig. 8

Supplementary Fig. 9

Supplementary Fig. 10

Supplementary Fig. 11

Supplementary Fig. 12

Supplementary Fig. 13
