# Supplementary material for: Distinct functions of transforming growth factor-β signaling in c-MYC driven hepatocellular carcinoma initiation and progression
Source: Cell Death Dis. 2021 Feb 19;12(2):200. doi: 10.1038/s41419-021-03488-z (PMC7895828; doi:10.1038/s41419-021-03488-z)
Supplement: Supplementary file 1 — Supplementary Table 1 [file 41419_2021_3488_MOESM1_ESM.docx]

**Supplementary Table 1.** Antibodies used for immunohistochemistry (IHC), immunofluorescence (IF), and Western blotting (WB)

| **IHC** | **Concentration** | **Company** | **Catalogue No.** |
| --- | --- | --- | --- |
| Cleaved Caspase 3 | 1:500 | Cell Signaling Technology | 9664 |
| CD45 | 1:50 | BD Biosciences | 550539 |
| F4/80 | 1:250 | Invitrogen | 2067198 |
| c-MYC | 1:100 | Abcam | Ab32072 |
| Ki67 | 1:150 | Thermo Fisher Scientific | RM-9106-S1 |
|  |  |  |  |
| **IF** | **Concentration** | **Company** | **Catalogue No.** |
| E-cadherin | 1:200 | BD Biosciences | 610404 |
| Vimentin | 1:100 | Cell Signaling Technology | 5741 |
|  |  |  |  |
| **WB** | **Concentration** | **Company** | **Catalogue No.** |
| β-Actin | 1:2000 | Cell Signaling Technology | 4970 |
| BCL-xL | 1:1000 | Cell Signaling Technology | 2764 |
| Cleaved Caspase 3 | 1:500 | Cell Signaling Technology | 9664 |
| c-MYC | 1:10000 | Abcam | Ab32072 |
| Cyclin B | 1:250 | Santa Cruz Biotechnology | SC-245 |
| Cyclin D1 | 1:2000 | Abcam | Ab134175 |
| GAPDH | 1:10000 | EMD Millipore | MAB374 |
| MCL-1 | 1:3000 | Cell Signaling Technology | 94296 |
| p-SMAD2/3 | 1:1000 | Cell Signaling Technology | 8828 |
| SMAD2/3 | 1:1000 | Cell Signaling Technology | 8685 |
| PCNA | 1:1000 | Cell Signaling Technology | 2586 |
| PMEPA1 | 1:1000 | Santa Cruz Biotechnology | sc-293372 |
|  |  |  |  |
| **2^nd^ Antibodies** | **Concentration** | **Company** | **Catalogue No.** |
| Goat anti-Mouse | 1:500 | Invitrogen | A11001 |
| Goat anti-Rabbit | 1:500 | Invitrogen | B2770 |
