# Supplementary material for: Distinct functions of transforming growth factor-β signaling in c-MYC driven hepatocellular carcinoma initiation and progression
Source: Cell Death Dis. 2021 Feb 19;12(2):200. doi: 10.1038/s41419-021-03488-z (PMC7895828; doi:10.1038/s41419-021-03488-z)
Supplement: Supplementary file 2 — Supplementary Table 2 [file 41419_2021_3488_MOESM2_ESM.docx]

**Supplementary Table 2.**  Sequences of the real-time PCR primers

| Genes | **Forward primer sequences**  **(5’-3’)** | **Reverse primer sequences**  **(5’-3’)** |
| --- | --- | --- |
| 18s rRNA | CGGCTACCACATCCAAGGAA | GCTGGAATTACCGCGGCT |
| *Mouse* |  |  |
| Angptl4 | CATCCTGGGACGAGATGAACT | TGACAAGCGTTACCACAGGC |
| Cdh1 | CAGTTCCGAGGTCTACACCTT | TGAATCGGGAGTCTTCCGAAAA |
| Cdh2 | AGGCTTCTGGTGAAATTGCAT | GTCCACCTTGAAATCTGCTGG |
| Clic4 | AAGGCCGGAAGTGATGGTG | GGTCAACGGTTGTGACACTGA |
| Cxcr4 | GACTGGCATAGTCGGCAATG | AGAAGGGGAGTGTGATGACAAA |
| Il-6 | TAGTCCTTCCTACCCCAATTTCC | TTGGTCCTTAGCCACTCCTTC |
| Il-11 | TGTTCTCCTAACCCGATCCCT | CAGGAAGCTGCAAAGATCCCA |
| Jag1 | CCTCGGGTCAGTTTGAGCTG | CCTTGAGGCACACTTTGAAGTA |
| Pmepa1 | TGGAGTTCGTGCAAATCGTG | TCCGAGGACAGTCCATCGTC |
| Pthrp | CATCAGCTACTGCATGACAAGG | GGTGGTTTTTGGTGTTGGGAG |
| Snai1 | CACACGCTGCCTTGTGTCT | GGTCAGCAAAAGCACGGTT |
| Tjp1 | GCCGCTAAGAGCACAGCAA | TCCCCACTCTGAAAATGAGGA |
| Twist1 | GGACAAGCTGAGCAAGATTCA | CGGAGAAGGCGTAGCTGAG |
| Vim | CGTCCACACGCACCTACAG | GGGGGATGAGGAATAGAGGCT |
| Zeb1 | GCTGGCAAGACAACGTGAAAG | GCCTCAGGATAAATGACGGC |
| *Human* |  |  |
| PMEPA1 | TGTCAGGCAACGGAATCCC | CAGGTACGGATAGGTGGGC |
