# Supplementary material for: Distinct functions of transforming growth factor-β signaling in c-MYC driven hepatocellular carcinoma initiation and progression
Source: Cell Death Dis. 2021 Feb 19;12(2):200. doi: 10.1038/s41419-021-03488-z (PMC7895828; doi:10.1038/s41419-021-03488-z)
Supplement: Supplementary file 4 — Supplementary Figure Legends [file 41419_2021_3488_MOESM4_ESM.docx]

**Supplementary Figure Legends**

**Supplementary Fig. 1.** Representative Western blot results showing upregulation of p-Smad2/3 in c-Myc/TGFβ1 tumors when compared with c-Myc/pT3 tumors. Gapdh was used as a loading control. At least four mice per group were analyzed.

**Supplementary Fig. 2. Inhibition of the TGFβ-SMAD signaling pathway accelerates c-Myc liver tumorigenesis in mice. (a)** Representative images of H&E, Ki67, and c-Myc immunohistochemistry in c-Myc/shLuciferase (shLuc), c-Myc/shSmad2 (shSmad2), c-Myc/shSmad3 (shSmad3), and c-Myc/shSmad4 (shSmad4) mouse HCCs. Scale bars: 200μm. **(b)** Representative images of GFP immunoflucence expression in c-Myc/shLuciferase (shLuc), c-Myc/shSmad2 (shSmad2), c-Myc/shSmad3 (shSmad3), and c-Myc/shSmad4 (shSmad4) mouse HCCs. Scale bars: 50μm. **(c)** Comparison of Ki67 positive cells percentages in four groups. Student t test was applied between each 2 groups for statistical analysis; no statistical significance was observed.

**Supplementary Fig. 3. Overexpression of Mcl-1 overrides the TGFβ1 suppression role on c-Myc tumor initiation. (a)** Study design. *FVB/N* mice were injected with c-Myc/Mcl-1/pT3/SB (N=5) or c-Myc/Mcl-1/TGFβ1/SB (N=5) plasmids. Mice were monitored and sacrificed when moribund. **(b)** Survival curve showing that Mcl-1 compensated the TGFβ1 suppression role on c-Myc tumor initiation. Kaplan-Meier method and log-rank test were applied. *P*= 0.1643. **(c)** Analysis of mouse livers injected with c-Myc/Mcl-1/TGFβ1 at 1-week (1W), 2-week (2W), and 3-week (3W) post injection. Representative images of H&E, Ki67 and c-Myc immunohistochemical staining were shown. Red arrows indicate Ki67 and c-Myc positive hepatocytes. Scale bars: 200μm for H&E, 100μm for Ki67 and c-Myc. **(d)** Representative images of Cleaved caspase-3 (C-C3) immunohistochemical staining in c-Myc/Mcl-1/pT3 and c-Myc/Mcl-1/TGFβ1 tumors. Quantification of C-C3 positive percentage showing no statistical difference between the two groups. Student *t* test was applied for statistical analysis.

**Supplementary Fig. 4. TGFβ1 activation does not affect cell growth in c-Myc murine tumor derived HCC cell lines. (a)** Comparison of cell proliferation percentages in HCC3-4 cells transfected with EGFP (N=3) or TGFβ1 (N=3). **(b)** Represented images and quantifications of cell colony formation assay in HCC3-4 cells transfected with EGFP (N=3) or TGFβ1 (N=3). Experiments were conducted three times. **(c)** Comparison of cell proliferation percentage in HCC4-4 cells transfected with EGFP (N=3) or TGFβ1 (N=3). **(d)** Represented images and quantifications of cell colony formation assay in HCC4-4 cells transfected with EGFP (N=3) or TGFβ1 (N=3). Experiments were conducted three times. Student *t* test was applied for statistical analysis. Abbreviation: N.S, not significant.

**Supplementary Fig. 5 Histological features of murine spleen 3 days after splenic injection.** Representative images of H&E and c-Myc staining of the spleens 3 days after splenic injection with HCC4-4 cells. Neoplastic lesions were observed within the spleens, showing positive staining of c-Myc. Scale bars: 200μm for 100X, 100μm for 200X. Abbreviations: T, tumor.

**Supplementary Fig. 6. TGFβ1 activation does not induce EMT in c-Myc murine HCCs.** **(a, b)** mRNA expression of TGFβ downstream target genes (*Snai1 Twist1,* and *Zeb1*) and EMT related genes (*Cdh1, Tjp1, Vim, Cdh2* and *S100a4*) in EGFP or TGF-β1 activated **(a)** HCC3-4 cell lines and **(b)** HCC4-4 cell lines. Student *t* test was applied for statistical analysis.

**Supplementary Fig. 7. TGFβ1 overexpression does not induce EMT in c-Myc/Mcl-1 murine HCCs.** Representative images of E-cadherin and Vimentin immunofluorescence staining in c-Myc/Mcl-1/pT3 and c-Myc/Mcl-1/TGFβ1 murine HCCs. No E-cadherin+ and Vimentin+ cells showed overlapping immunoreactivity, indicating the absence of EMT in the c-Myc/Mcl-1/TGFβ1 HCCs. Scale bar: 75μm.

**Supplementary Fig. 8. mRNA levels of the three TGFβ isoforms do not show negative correlation with mRNA expression of epithelial markers in human HCCs. (a)** Spearman’s correlation of *CDH1* mRNA expression and *TGFB1*, *TGFB2* and *TGFB3* mRNA levels in human HCCs. **(b)** Spearman’s correlation of *TJP* mRNA expression and *TGFB1*, *TGFB2* and *TGFB3* mRNA levels in human HCCs. Images were obtained from the public cBioPortal site (https://www.cbioportal.org/)

**Supplementary Fig. 9. TGFβ1 modulates the tumor microenvironment. (a)** Representative images and quantification of Cd45 immunohistochemistry in EFGP and TGFβ1 overexpressing c-Myc liver tumors. Red triangles indicate Cd45 positive lymphocytes. **(b)** Representative images and quantification of F4/80 immunohistochemical staining in EFGP and TGFβ1 overexpressing c-Myc liver tumors. Scale bars: 200 μm.

**Supplementary Fig. 10. The TGFβ-SMAD signaling pathway is activated in human HCCs. (a)** *Smad2, Smad3，Smad4, Smad7, TGFβ1, TGFβ2,* and *TGFBR1,* mRNA levels changes in HCC patients across different cohorts. Data were obtained from Oncomine (https://www.oncomine.org/resource/) and were presented as log2 fold change. *P*<0.05 for each group. **(b)** Protein expression of SMAD4 and c-MYC showed a tendency of positive correlation in TCGA LIHC dataset. Each dot in the figure indicates one HCC sample. Red dots indicate HCC samples that have both c-MYC positive and SMAD positive expression, while blue dots indicate HCC samples that have both c-MYC negative and SMAD negative expression. Data were obtained from https:// tcpaPortal.org.

**Supplementary Fig. 11. TGFβ regulates cell apoptosis in c-Myc murine HCCs.** Western blot results showing upregulation of p-Smad2/3, anti-apoptotic Mcl-1, and Bcl-xL, and cell cycle regulators Ccnb and Ccnd1, in c-Myc/TGFβ1 tumors when compared with c-Myc/pT3 tumors. Gapdh was used as a loading control. Western blot bands for c-Myc, Smad2/3, p-Smad2/3 and Gapdh were the same as in Supplementary Fig. 1. Gapdh was used as a loading control.

**Supplementary Fig. 12. Inactivation of TGFβ downstream EMT target genes in c-MYC amplified human HCCs.** Downregulation of TGFβ downstream target genes (*CREBBP, EP300, APC, SKIL* and *MAP2K1*) in c-MYC amplified (MYC Amp; n =64) HCCs than that in c-MYC non-mutated (MYC Wt; n =293) HCCs. Student *t* test was applied for statistical analysis, **, *P* < 0.01, *** *P*<0.001, ****, *P* <0.0001.

**Supplementary Fig. 13. Increased expression of *PMEPA1* in human HCCs according to the TCGA dataset. (Upper panel)** Box-Whisker plot showing *PMEPA1* expression level in normal liver tissues (Normal; n=50) and primary liver tumors (HCCs; n=371) samples in the TCGA dataset. Data were presented as transcript per million (TPM). Student’s *t* test was applied for statistical analysis, *P* <0.0001. **(Lower panel)** Detailed information for interquartile range of minimum, 25th percentile, median, 75th percentile and maximum TPM values. Data were obtained from UALCAN (http://ualcan.path.uab.edu/index.html).
