# Supplementary figures and images for: Distinct functions of transforming growth factor-β signaling in c-MYC driven hepatocellular carcinoma initiation and progression
Source: Cell Death Dis. 2021 Feb 19;12(2):200. doi: 10.1038/s41419-021-03488-z (PMC7895828; doi:10.1038/s41419-021-03488-z)

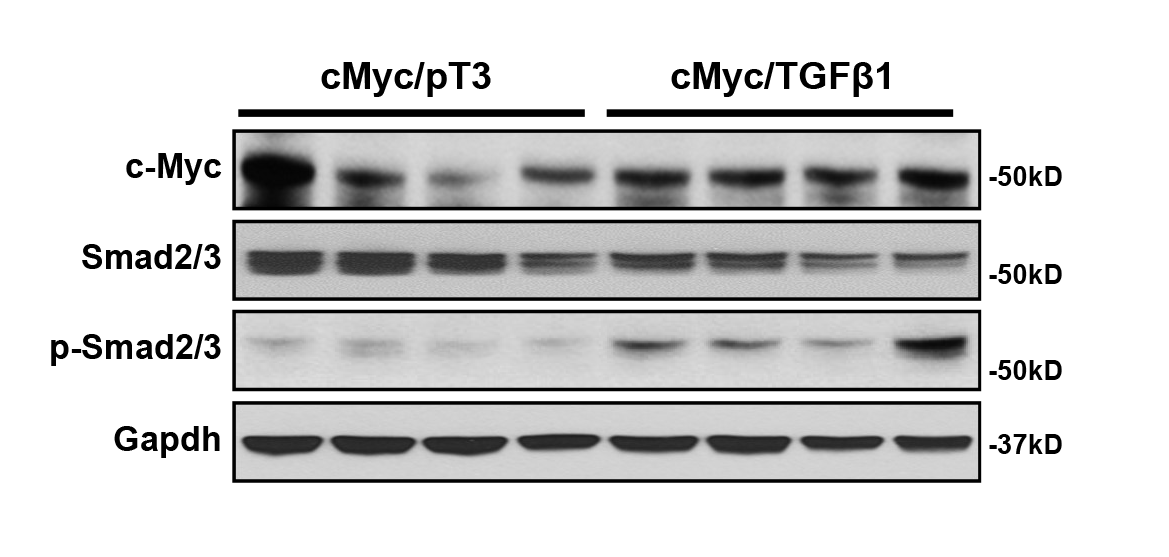

Supplement: Supplementary file 5 — Supplementary Fig. 1 [file 41419_2021_3488_MOESM5_ESM.tif]

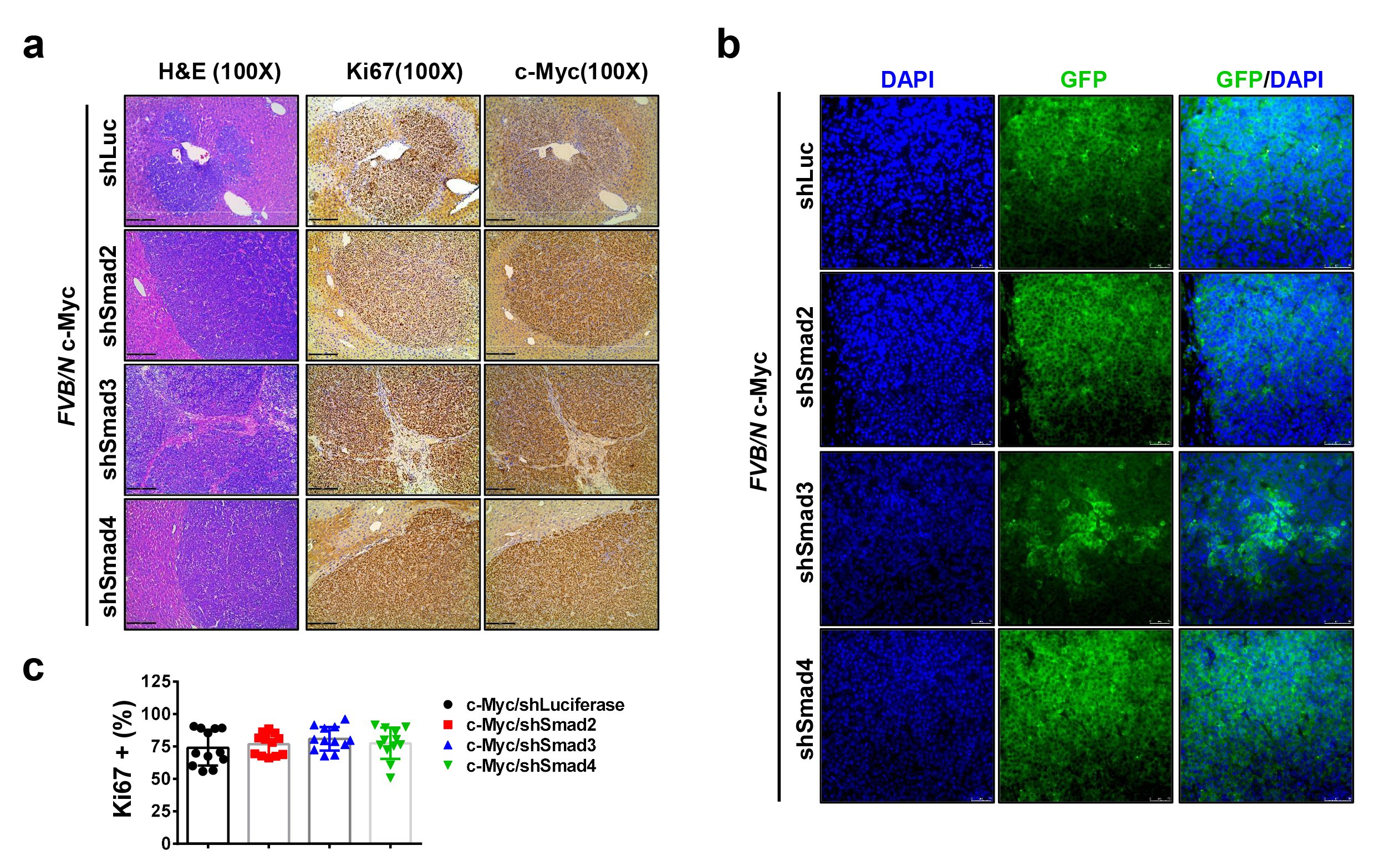

Supplement: Supplementary file 6 — Supplementary Fig. 2 [file 41419_2021_3488_MOESM6_ESM.tif]

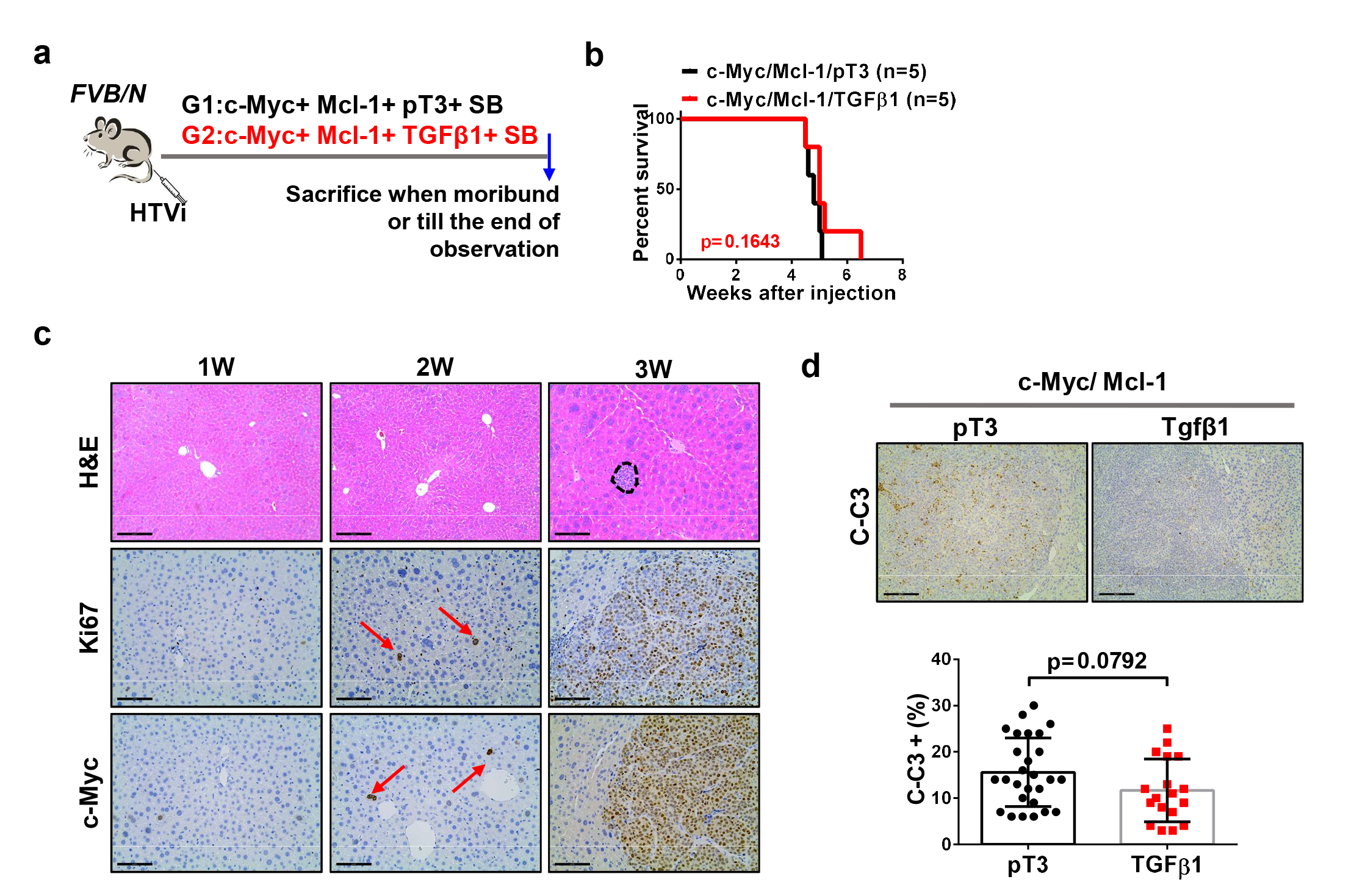

Supplement: Supplementary file 7 — Supplementary Fig. 3 [file 41419_2021_3488_MOESM7_ESM.tif]

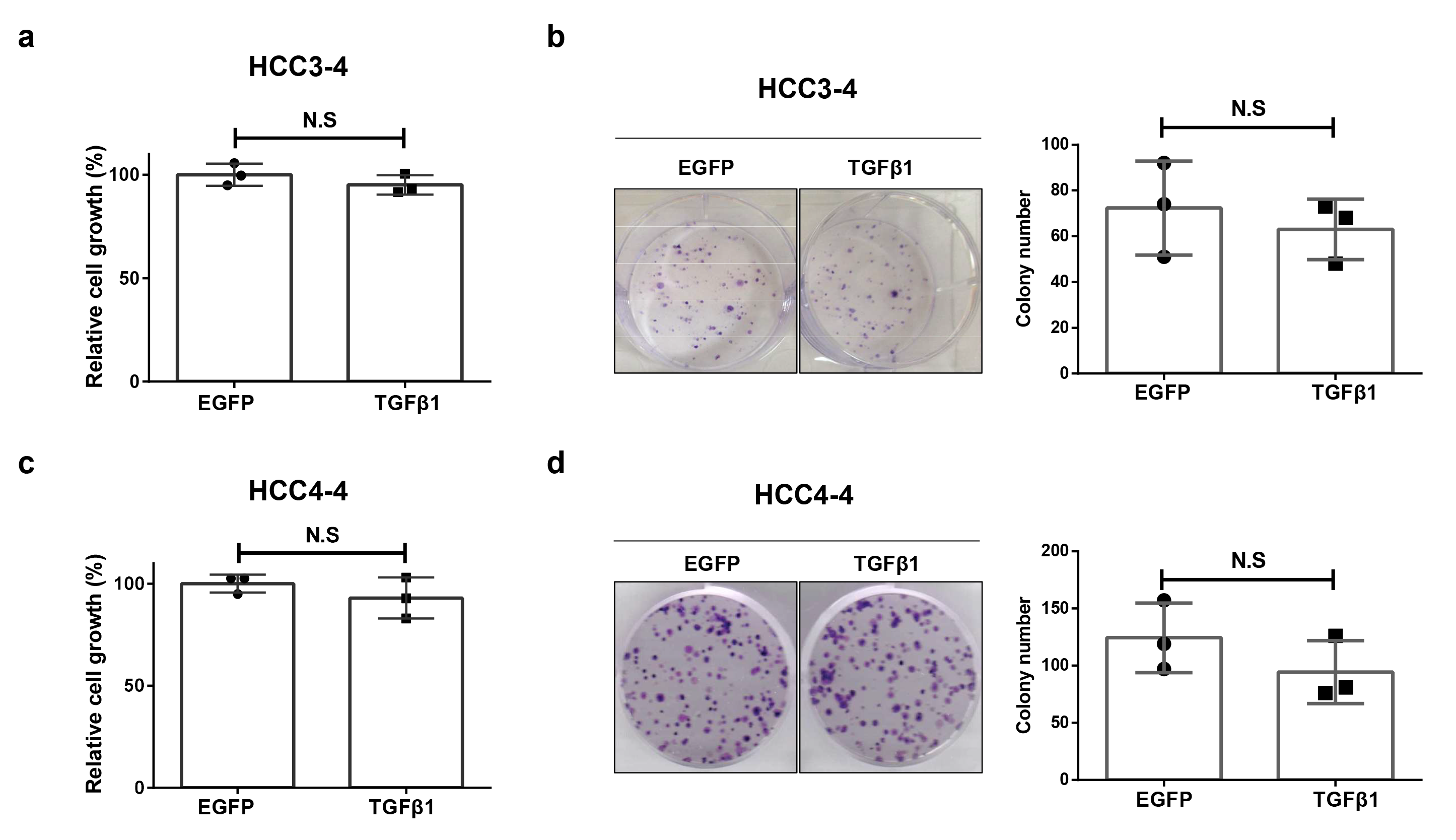

Supplement: Supplementary file 8 — Supplementary Fig. 4 [file 41419_2021_3488_MOESM8_ESM.tif]

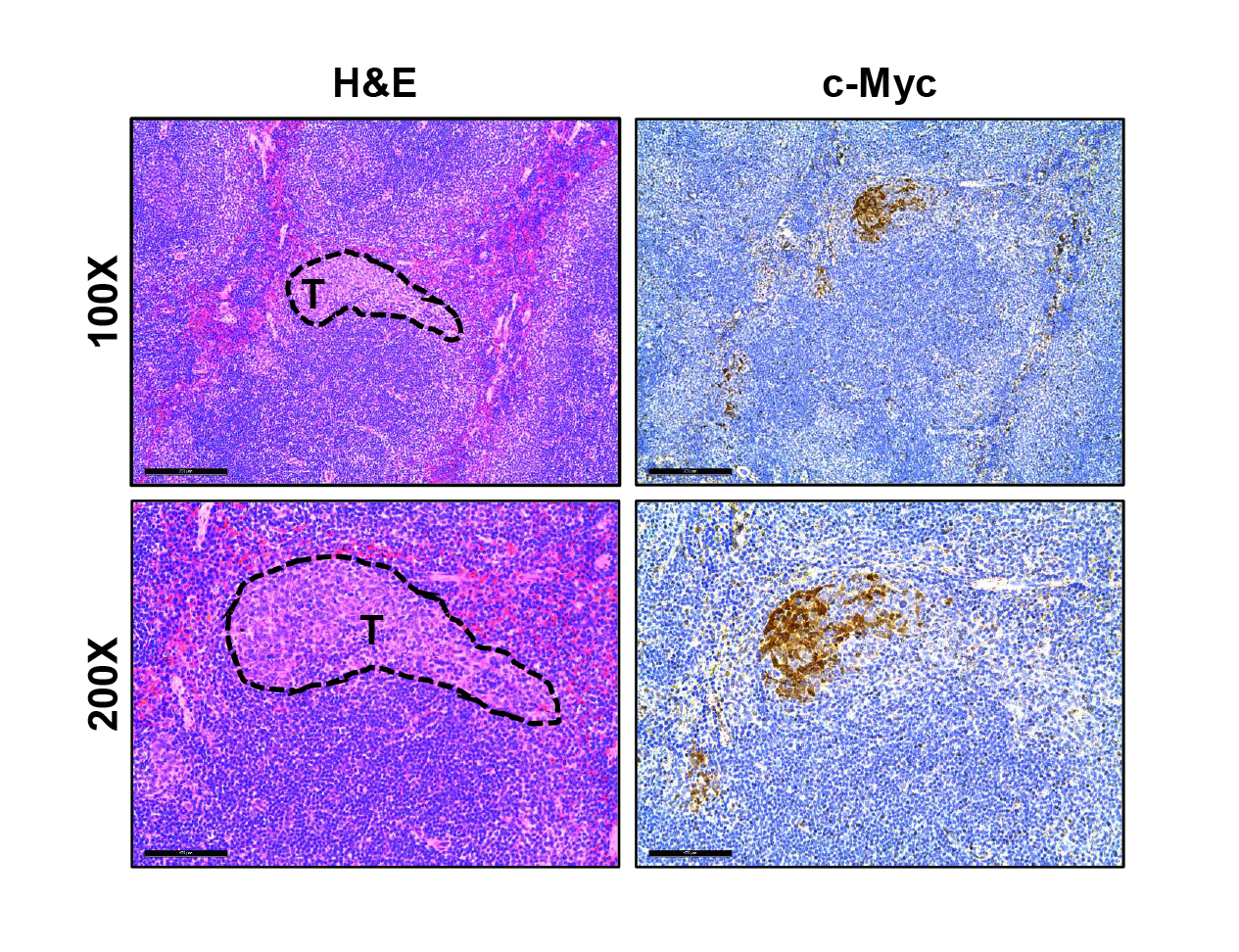

Supplement: Supplementary file 9 — Supplementary Fig. 5 [file 41419_2021_3488_MOESM9_ESM.tif]

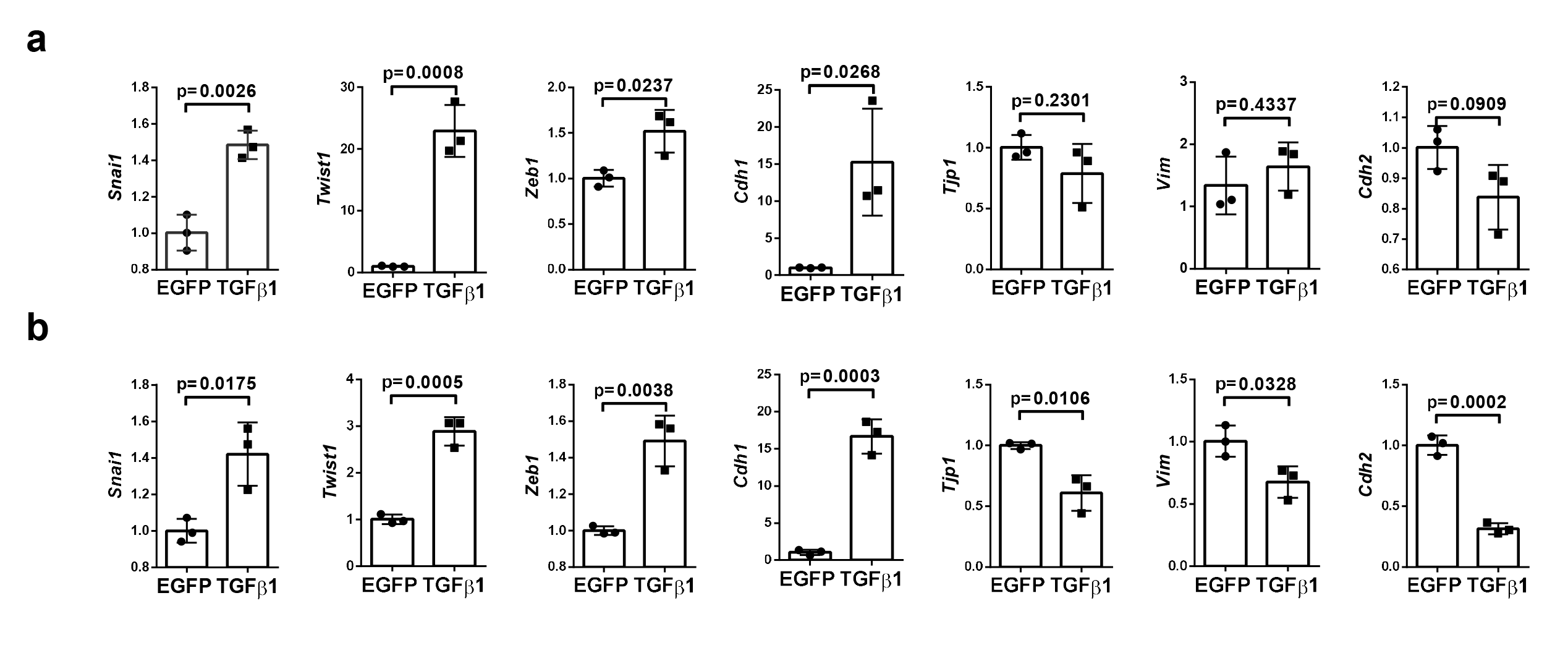

Supplement: Supplementary file 10 — Supplementary Fig. 6 [file 41419_2021_3488_MOESM10_ESM.tif]

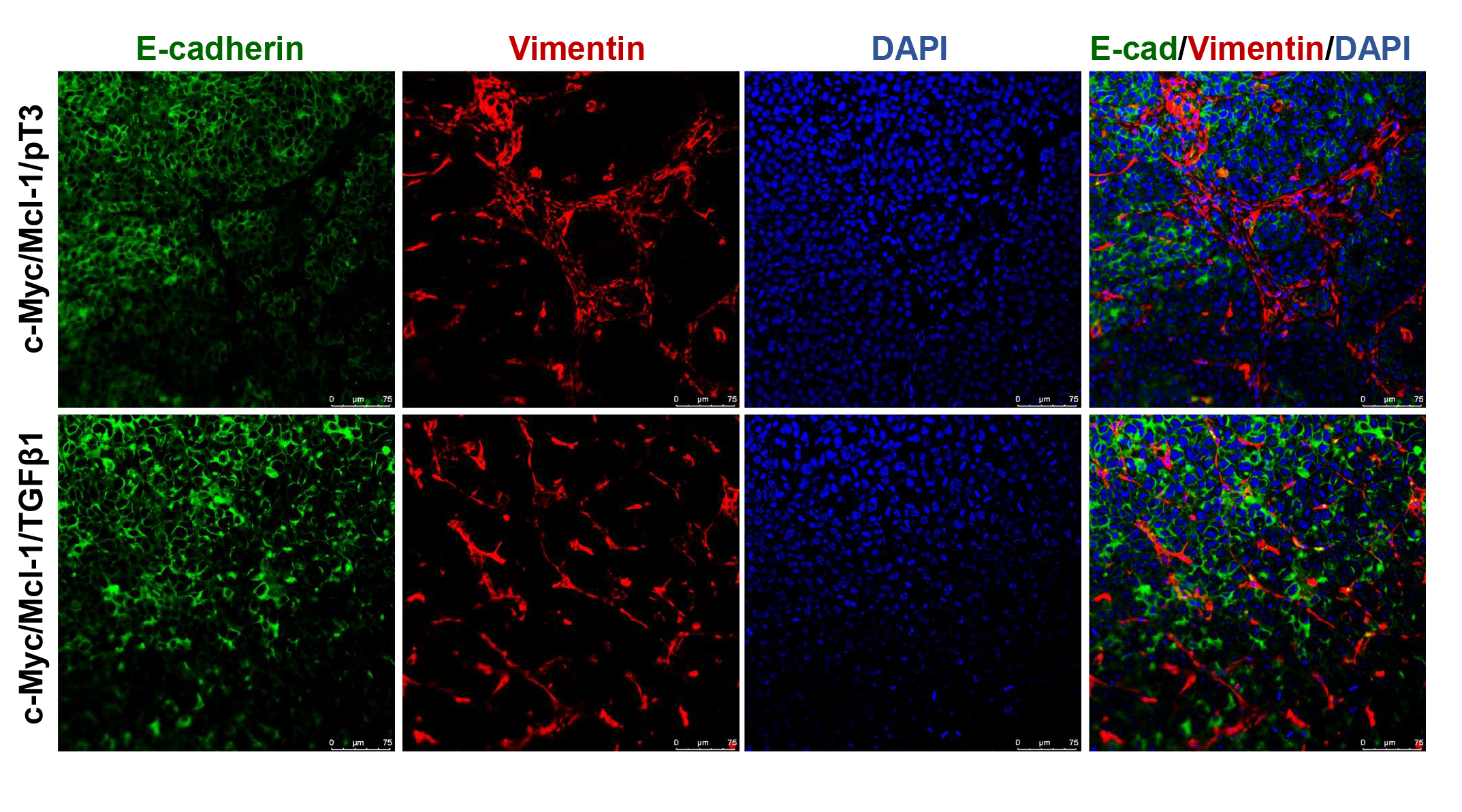

Supplement: Supplementary file 11 — Supplementary Fig. 7 [file 41419_2021_3488_MOESM11_ESM.tif]

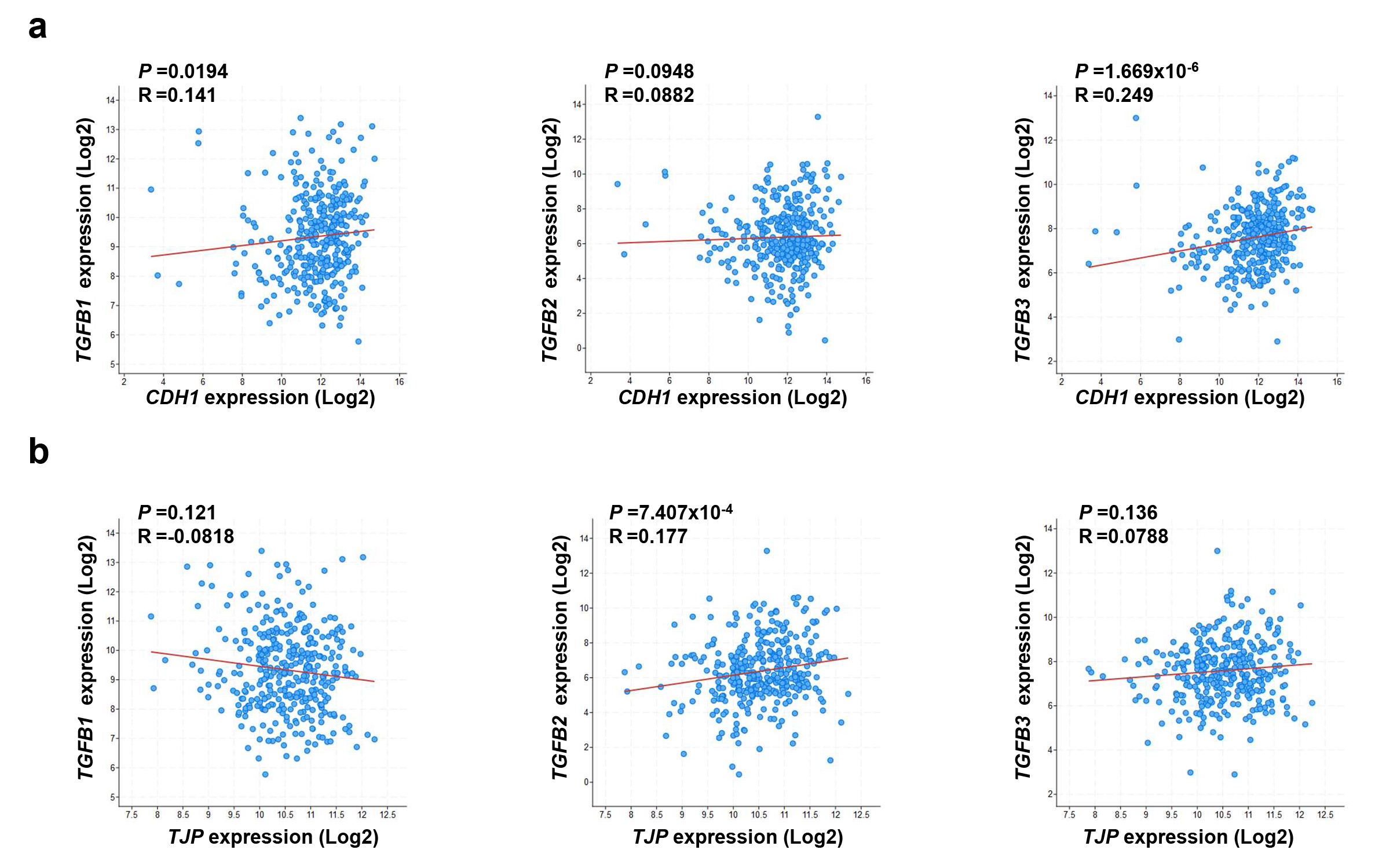

Supplement: Supplementary file 12 — Supplementary Fig. 8 [file 41419_2021_3488_MOESM12_ESM.tif]

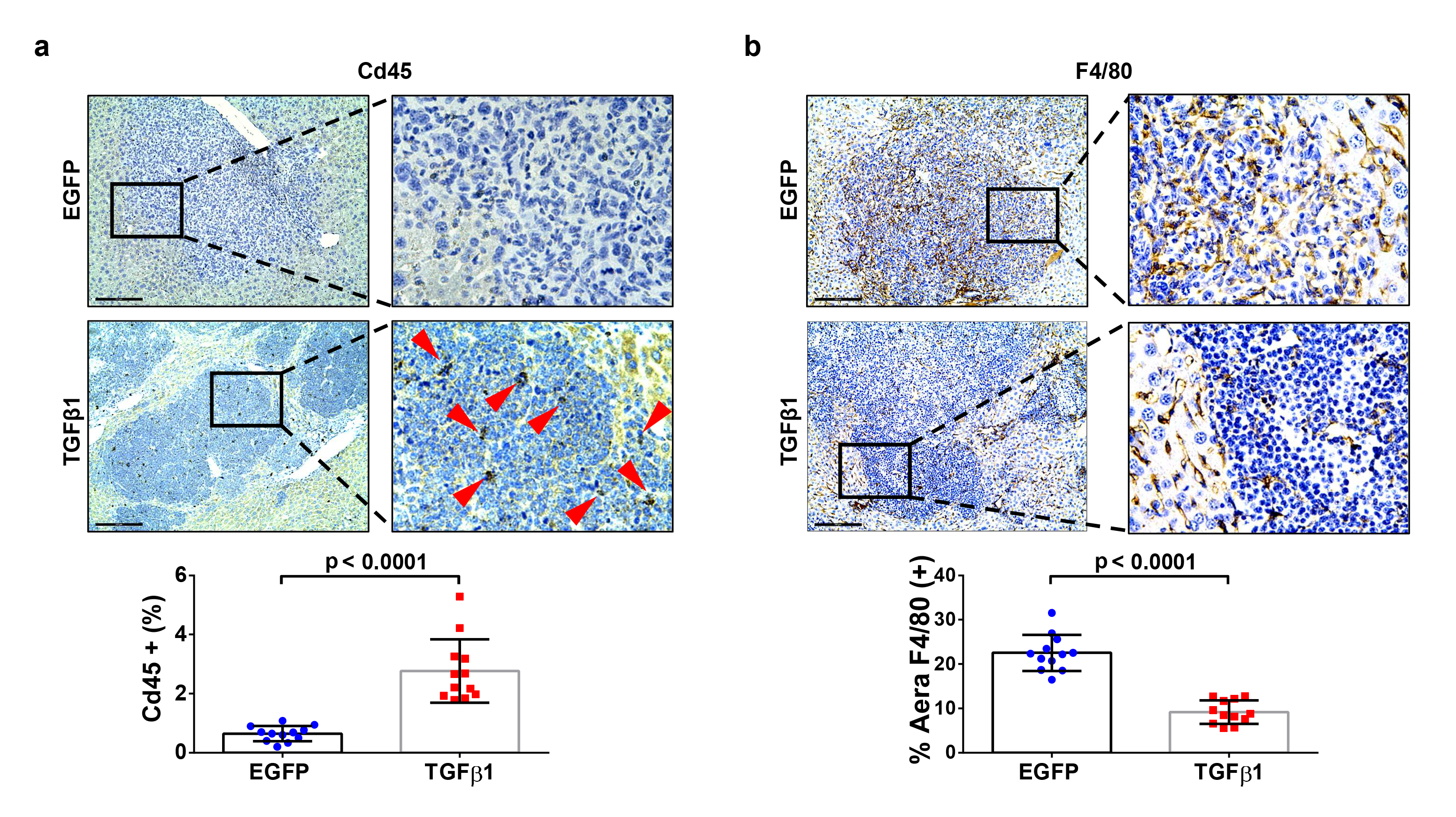

Supplement: Supplementary file 13 — Supplementary Fig. 9 [file 41419_2021_3488_MOESM13_ESM.tif]

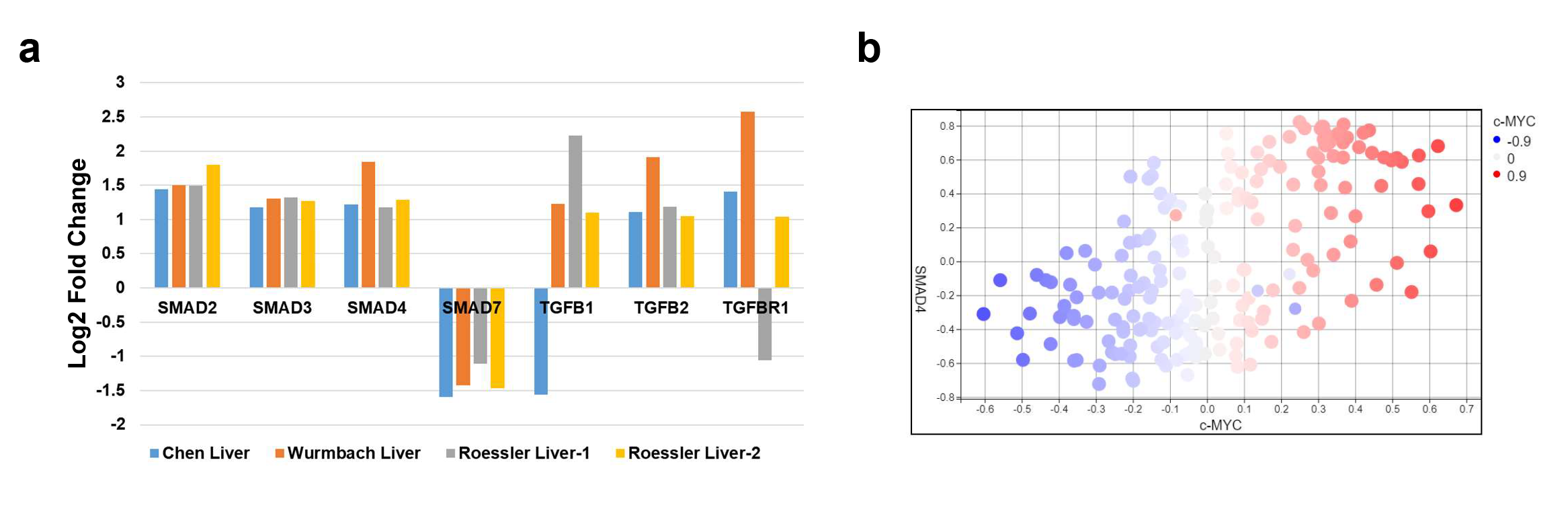

Supplement: Supplementary file 14 — Supplementary Fig. 10 [file 41419_2021_3488_MOESM14_ESM.tif]

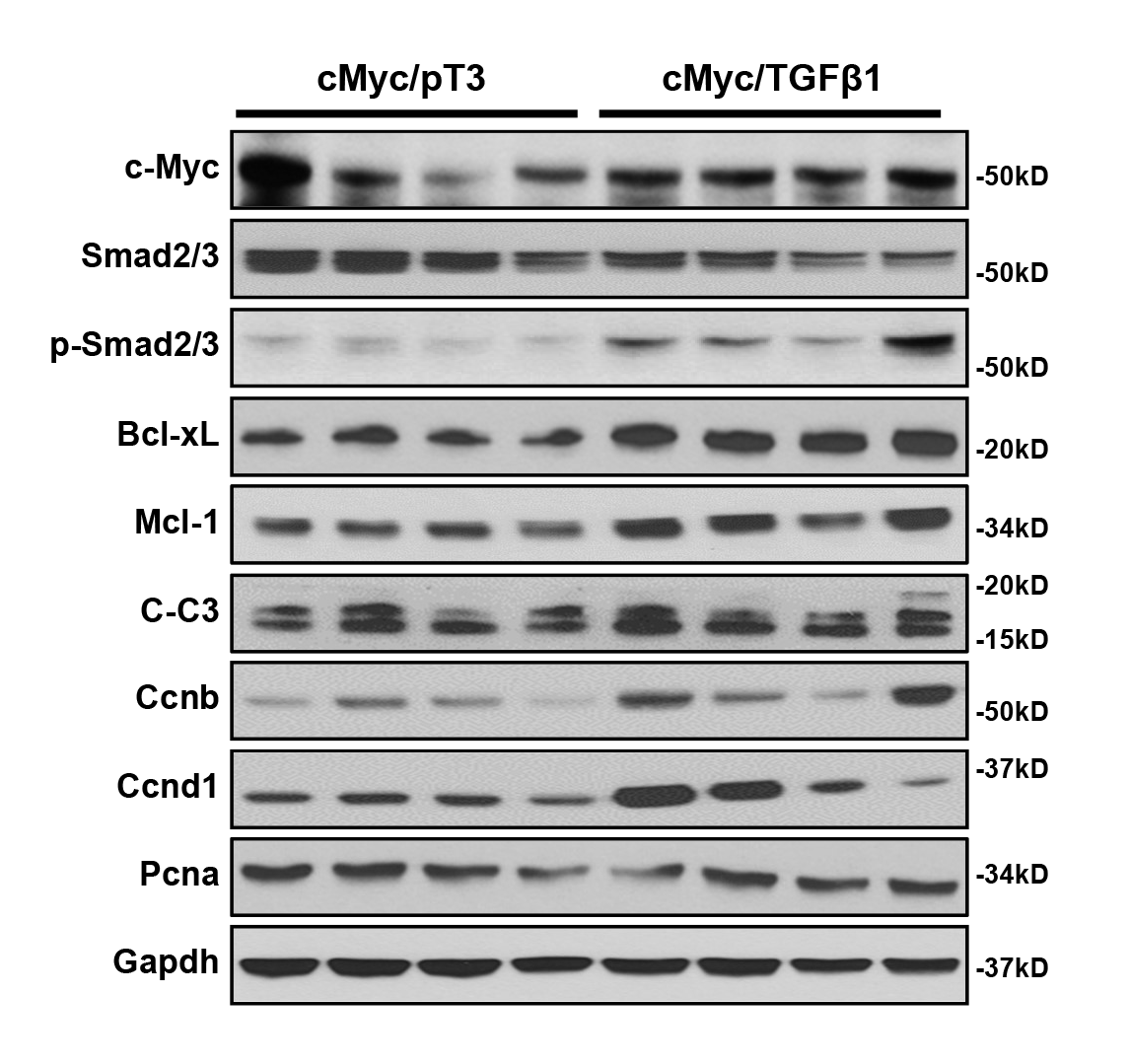

Supplement: Supplementary file 15 — Supplementary Fig. 11 [file 41419_2021_3488_MOESM15_ESM.tif]

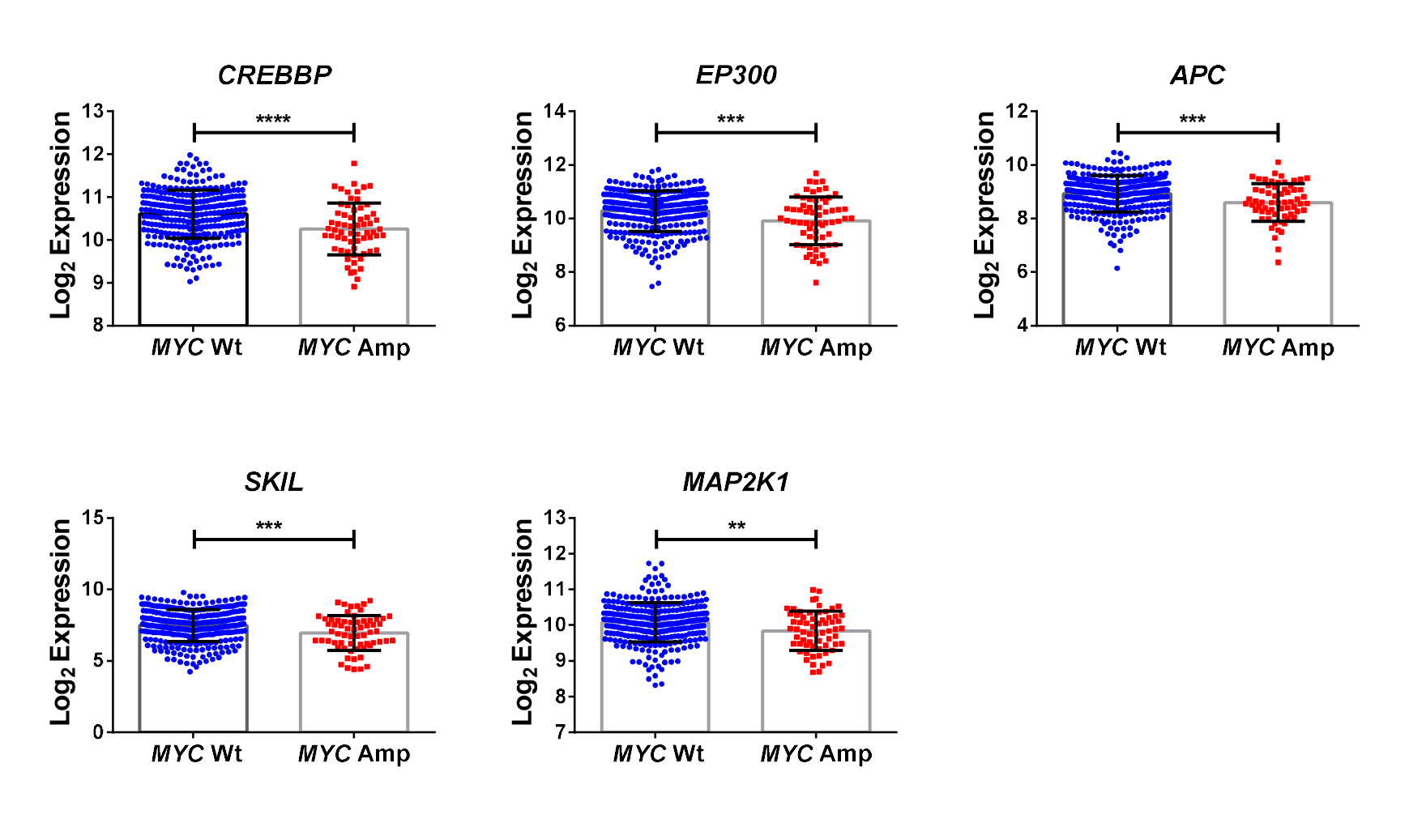

Supplement: Supplementary file 16 — Supplementary Fig. 12 [file 41419_2021_3488_MOESM16_ESM.tif]

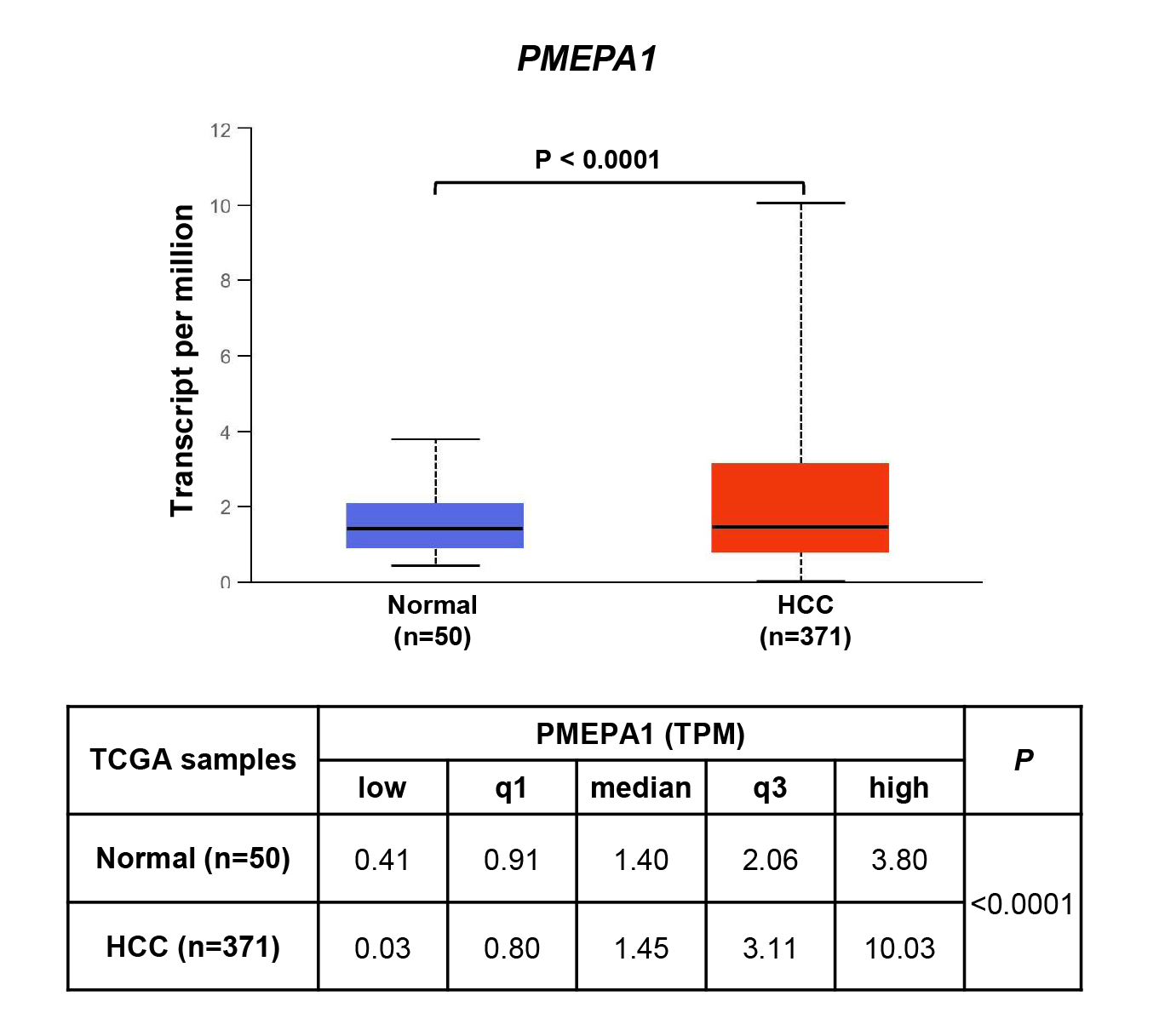

Supplement: Supplementary file 17 — Supplementary Fig. 13 [file 41419_2021_3488_MOESM17_ESM.tif]
